# An Attempt at a Unified Theory of the Neocortical Microcircuit in Sensory Cortex

**DOI:** 10.3389/fncir.2020.00040

**Published:** 2020-07-28

**Authors:** Max Bennett

**Affiliations:** Independent Researcher, New York, NY, United States

**Keywords:** sequence memory, neocortex, neocortical theory, prediction, hierarchical temporal memory (HTM), chunking and cognition, working memory, delay activity

## Abstract

The neocortex performs a wide range of functions, including working memory, sensory perception, and motor planning. Despite this diversity in function, evidence suggests that the neocortex is made up of repeating subunits (“macrocolumns”), each of which is largely identical in circuitry. As such, the specific computations performed by these macrocolumns are of great interest to neuroscientists and AI researchers. Leading theories of this microcircuit include models of predictive coding, hierarchical temporal memory (HTM), and Adaptive Resonance Theory (ART). However, these models have not yet explained: (1) how microcircuits learn sequences input with delay (i.e., working memory); (2) how networks of columns coordinate processing on precise timescales; or (3) how top-down attention modulates sensory processing. I provide a theory of the neocortical microcircuit that extends prior models in all three ways. Additionally, this theory provides a novel working memory circuit that extends prior models to support simultaneous multi-item storage without disrupting ongoing sensory processing. I then use this theory to explain the functional origin of a diverse set of experimental findings, such as cortical oscillations.

## Introduction

Understanding the exact computations performed by the mammalian neocortex has been a “Holy Grail” of Neuroscience for over 100 years. This is in part inspired by the fact that the only known unique attribute of the human brain in comparison to other mammals is the relative size of our neocortex (Herculano-Houzel, 2009). Furthermore, there is broad consensus that the neocortex is where working memory is stored (Goldman-Rakic, 1995), where the neural correlates of consciousness are contained (Koch et al., 2016), where facial recognition occurs (Kanwisher et al., 1997), where music perception occurs (Zatorre et al., 2007), where “cognitive control” happens (Miller, 2000), where complex motor tasks such as playing a sport or musical instrument are learned (Papale and Hooks, 2018), where decision making occurs (Kable and Glimcher, 2009), and much more.

And yet, despite this astronomically wide range of functions, the neocortex seems to be made up of repeating subunits called “macrocolumns,” each of which contains the same types of neurons, connectivity, and firing properties (Mountcastle, 1978). This observation has led to the hypothesis that the neocortex is just a repeated replication of the *exact same* microcircuit and that there was an evolutionary benefit to this duplication (Mountcastle, 1978; Douglas et al., 1989; Douglas and Martin, 2004; Thomson and Lamy, 2007; George and Hawkins, 2009; Harris and Mrsic-Flogel, 2013). Additional support for this can be seen in rerouting studies, whereby rerouting visual input to auditory cortex seems to convert auditory cortex into a visual cortex, suggesting that the only difference between these two regions is the input they receive and not the computations they perform (von Melchner et al., 2000). This is further supported by the fact that the human neocortex increased in size by almost 3-fold over just the last 3 million years of human evolution (Du et al., 2018), a time frame likely too fast for any new circuitry to emerge other than a duplication of existing circuits. This hypothesis suggests that the only difference between any two areas of the neocortex is the inputs it receives, and the location it sends its outputs—the actual computations themselves are the same. If true, this would dramatically reduce the theoretical complexity of understanding the human neocortex from trying to understand the connectivity of ~20 billion neurons and ~100 trillion synapses, to simply trying to understand the far fewer number of neurons and synapses within the “neocortical microcircuit” that is being duplicated.

Despite the above evidence, there are legitimate challenges to the hypothesis that the neocortex implements a repeated canonical microcircuit (further elucidated in the “Discussion” section). The most notable differences exist between the “frontal cortex” and “sensory cortex” (Fukutomi et al., 2018). As such, most models of the neocortical microcircuit, including this one, focus their efforts on unraveling the alleged microcircuit within the *sensory* cortex.

There are four leading computational frameworks of the neocortical microcircuit within the sensory cortex: predictive coding, hierarchical temporal memory (HTM), bayesian inference, and Adaptive Resonance Theory (ART). These have all been broadly categorized as “predictive processing framework.” All these predictive processing frameworks share two essential features. First, they all assume that the purpose of the sensory cortex is to predict its sensory input. Second, they all assume that the neocortex performs computations, at least in part, hierarchically—whereby the outputs of lower-order regions are provided as inputs to higher-order regions. Although there is a broad consensus on these two features, there are notable differences between each framework, which I describe in more detail within the “Relationship to Previous Models” section. This work integrates and extends ideas from all four of these frameworks, but is built almost directly on top of HTM. HTM is uniquely attractive in that it models sequence and object learning using only Hebbian plasticity, whereas other models tend to require less biologically plausible learning mechanisms (see “Relationship to Previous Models” section).

However, three key elements are missing from prior HTM models that I seek to extend in this article. First, the neocortex can learn sequences even when input elements are separated by long time intervals (e.g., seconds to minutes), even though short term synaptic plasticity can only occur on the timescale of <100 ms (Markram et al., 1997). For example, say “A,” pause 5 s, say “B,” pause 5 s, say “C,” and then ask someone to repeat the sequence, and anyone can do so effortlessly. In other words, the neocortex can store elements of a sequence in working memory. However, prior HTM models have not incorporated working memory. Second, it appears evident that different macrocolumns coordinate processing together at precise timescales, otherwise it would be impossible for macrocolumns organized in a hierarchy to integrate information accurately. However, I am unaware of a neural circuit model that explains how such precisely timed coordination occurs. Third, prior HTM models do not explicitly incorporate attention.

As such, I seek to present a model that can perform the same computations of prior HTM models, but can also: (1) perform working memory and connect sequences separated by long time intervals; (2) coordinate its activity and processing with other macrocolumns and structures on extremely precise time intervals; and (3) can be modulated by attention. I will go on to show how this model directly explains a wide range of seemingly disparate experimental observations about the neocortex.

My approach will be to start with a basic overview of the overall organizational principles of neocortical neurons, macrocolumns, and thalamocortical networks. I will go on to assign specific computational roles to individual types of neurons within a macrocolumn. I will then incorporate input from the frontal cortex into these macrocolumns; and lastly, I will go on to show how networks of these macrocolumns can recognize and learn objects and sequences.

## An Overview of The Structure of Sensory Cortex

### The Structure of a Single Excitatory Neocortical Neuron

To model the computations within canonical neocortical microcircuit, we must first model the computations of a single neuron. Most of the excitatory neurons within the neocortex are pyramidal neurons, with many apical and basal dendritic segments. Most synapses on a pyramidal neuron are not proximal to the soma but rather found far away from soma on basal dendrites or apical dendrites (“distal synapses”). Presynaptic firing at distal synapses has very little effect on somatic membrane potential (Antic et al., 2010; Major et al., 2013). However, if coincident distal synapses fire simultaneously, a dendritic branch will fire its own NMDA dendritic spike, which can cause a sustained subthreshold (no action potential) depolarization at the soma (Antic et al., 2010; Major et al., 2013). Because dendritic segments spike on their own, neurons can learn new patterns without somatic action potentials. If a pattern of coincident input occurs frequently, dendritic spikes will lead to long term potentiation. Hence, neurons can passively learn to recognize patterns without firing somatic action potentials.

**Figure 1:**
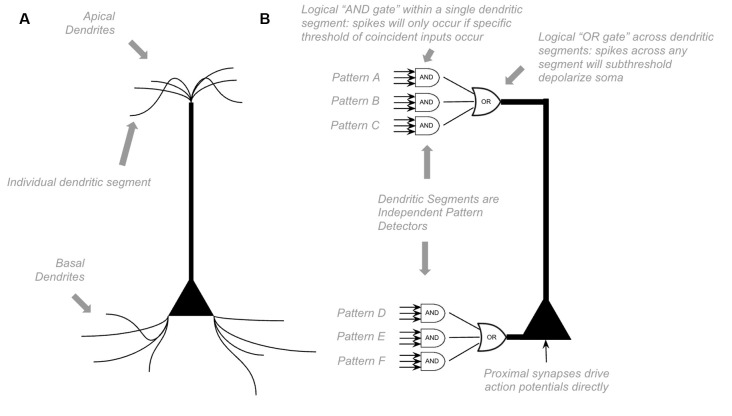
Proposed Computations performed by a single pyramidal neuron. **(A)** Visual depiction of morphology of a standard pyramidal neuron. **(B)** Proposed computations performed by inputs to distal dendritic segments vs. inputs to proximal dendritic segments. Dendritic segments are independent pattern detectors. Similar to that proposed by Hawkins et al. (2010) and Hawkins and Ahmad (2016). See text for details.

Unlike other models, HTM models incorporate the above dynamics directly (Hawkins et al., 2010; Hawkins and Ahmad, 2016). Each dendritic segment of an HTM model neuron is its own independent pattern recognizer. Dendritic spikes can be thought of as a logical “and” operation on its learned patterns, only firing a spike if a specific threshold of coincident presynaptic neurons fire. Whereas somatic depolarization effectively performs a logical “or” operation on each dendritic pattern recognizer (see Figure 1). Of course, in actuality, there is a non-linear summation of these presynaptic inputs, instead of an explicit “and” operation, but this can still be conceptually approximated as a logical “and.”

HTM models propose that pyramidal neurons always exist in one of three states: inactive, predictive, and active (Hawkins et al., 2010). In an inactive state, the neuron is highly polarized. In an active state, a neuron is firing action potentials. In a predictive state, a neuron is subthreshold depolarized. The computational purpose of this predictive state is that if a proximal synapse has a presynaptic action potential, neurons in predictive states will fire *before* neurons in inactive states. In parts of the neocortex with extensive lateral inhibition, this will lead to neurons that were in a predicted state firing action potentials, but those that were in inactive states *not* firing at all because they get rapidly inhibited before they have a chance to depolarize. This dynamic is an essential computational motif in HTM (Hawkins and Ahmad, 2016).

### The Structure of a Single Macrocolumn

The sensory neocortex has six distinct layers of neurons, each containing different types of neurons with unique connectivity. Much of the connectivity of the neurons within a given area of the sensory cortex is horizontally contained within a 300–600 micron wide column, although spanning vertically across all six layers (Mountcastle, 1997). This “cortical macrocolumn” of local horizontal connectivity has been proposed to be the canonical neocortical microcircuit (Mountcastle, 1978, 2003; Rakic, 1988; Tsunoda et al., 2001). The human neocortex is thought to be made up of over a million such macrocolumns (Sporns et al., 2005).

**Figure 2:**
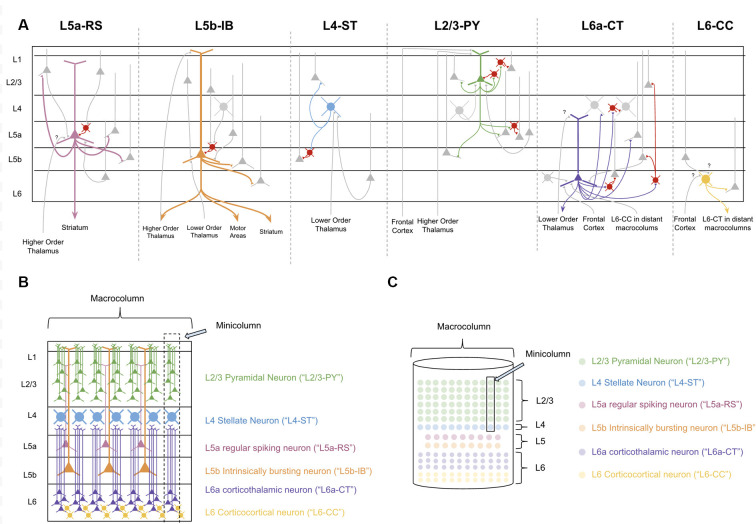
The Canonical neocortical microcircuit. **(A)** The main input/output connectivity of excitatory neuron types within microcircuit. Gray neurons denote excitatory neurons that are either afferent or efferent partners with neuron type in question. Red neurons denote inhibitory interneurons. Question marks (“?”) denote hypothesized but unverified connectivity. Triangles denote synapses. See Supplementary Table S1 for references on connectivity. **(B)** Visualization of morphology and laminar distribution of the main types of excitatory neurons within the neocortical microcircuit. Note that there are several known excitatory neuron types not depicted here, including L6b corticothalamic neurons, L6 corticoclaustral neurons, and L2/3 corticocortical pyramidal neurons. Also note that L6-CC neurons are highly varied in morphology, but for simplicity they are only the “star shaped” morphology is depicted here. **(C)** A simplified visual model of the neocortical microcircuit. This article will use this simplified visual model to conceptually articulate the computations performed by the macrocolumn.

In order to decipher the computations within a macrocolumn, we must interpret the observed connectivity of each of these types of neurons. Excitatory neurons within a macrocolumn can be categorized into nine main groups based on electrophysiology, morphology, and connectivity (see Figure 2A and Supplementary Table S1).

L4 and L2/3 of a macrocolumn can also be subdivided vertically into ~80–100 minicolumns, each of which is about 50 microns wide (Peters and Yilmaz, 1993; Mountcastle, 1997). In our model macrocolumn (see Figures 2B,C) there is one L4 stellate cell per minicolumn and many L2/3 cells within a minicolumn. Cells within L5 and L6 are not mapped to a specific minicolumn, but rather perform computations across the entire macrocolumn.

### The Structure of Thalamocortical Networks

In order to understand how the sensory neocortex performs its many functions, we must also consider the thalamus, which is the primary subcortical structure providing input to the neocortex (Sherman and Guillery, 2006). The thalamus relays information from sensory organs, such as the eyes and ears, to the neocortex, as well as passing information in-between areas of the neocortex (Sherman and Guillery, 2006).

The thalamus is primarily made up of excitatory thalamocortical relay neurons. Recent experimental studies have shown that there are three categories of these thalamocortical relay neurons within sensory thalamus: “Core Neurons,” “Multiareal Matrix Neurons,” and “Local Matrix Neurons” (Clascá et al., 2012). Each of these has different connectivity with the neocortex. Core neurons project directly to L4-ST neurons. Local Matrix Neurons project to layer 1 within a single level of the cortical hierarchy. Multiareal matrix neurons project to L5a, L1, and L3 across different levels of the hierarchy. Multiareal matrix neurons are also the only one of the three types of relay neurons that project directly to the striatum and the amygdala.

**Figure 3:**
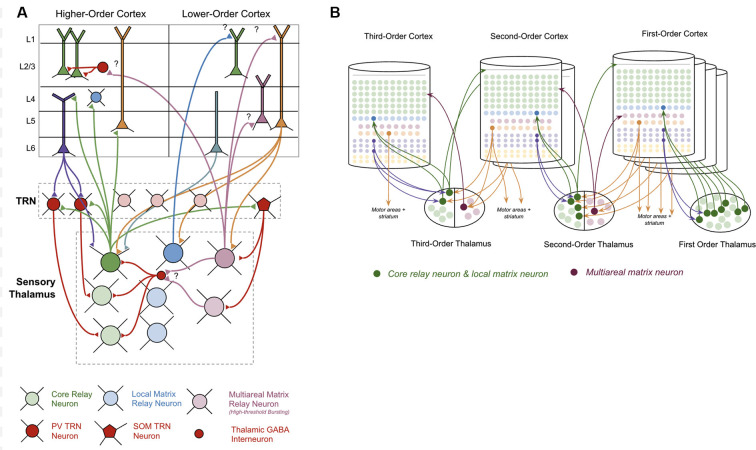
Connections between thalamus and neocortex. **(A)** Neuron types within the thalamus and their connectivity with neuron types within the neocortex. Question marks (“?”) denote hypothesized but unverified connectivity. Triangles denote synapses. See Supplementary Table S1 for references on connectivity. **(B)** A simplified visual model of a hierarchical thalamocortical network. Note that core relay neurons and their projections to higher-order cortex, and local matrix neurons and their projections to lower-order cortex are both depicted graphically as a single neuron. I do this both for visual simplicity, but also because in this article I hypothesize they communicate the same signals forward and backwards across the hierarchy. For visual simplicity, I only depict inputs to multiareal matrix neurons from some macrocolumns, but in the proposed model all lower order macrocolumns will project to these neurons.

The thalamus is organized hierarchically, with “first order relay nuclei” passing information directly from peripheral senses (e.g., sight, sound, touch) to “first order neocortex,” while higher-order relay nuclei pass information between different levels of neocortex within the hierarchy (see Figure 3B). Early on in this hierarchy, thalamic nuclei are separated by modalities, with separate nuclei for vision, audition, and somatosensation (Sherman and Guillery, 2006).

The nature of the connectivity between the thalamus and macrocolumns provides clues as to the computations that are being performed in thalamocortical networks (see Figure 3A). L5b-IB neurons provide *driving* input (synapses close to soma) to core relay neurons that project to L4 in other “higher-order” macrocolumns (Deschênes et al., 1994; Rouiller and Welker, 2000; Reichova and Sherman, 2004; Groh et al., 2008; Llano and Sherman, 2008; Theyel et al., 2009; Harris and Mrsic-Flogel, 2013; Sherman, 2017). These higher-level macrocolumns seem to repeat the same pattern of relaying their L5b-IB output through even higher-level thalamic relays to even higher-level macrocolumns. There is also evidence to suggest that L5b-IB neurons provide driving input to local matrix neurons, which project *back* to L1 in the originating macrocolumn (Ohno et al., 2012; Pouchelon et al., 2014).

In contrast to L5b-IB neurons, L6a-CT neurons provide *modulatory* input (synapses far away from the soma) back to the relay neurons that projected to L4-ST neurons in a given macrocolumn (Reichova and Sherman, 2004; Thomson, 2010; Sherman, 2017). These L6a-CT projections are generally thought of as the origin of “top-down” signals (Douglas and Martin, 2004). They are not able to drive action potentials in thalamic relay neurons on their own, but they can increase the firing rate of an already activated thalamic relay neuron *via* these modulatory synapses or put them into a subthreshold predictive state.

Surrounding the thalamus is a thin sheet of inhibitory neurons called the thalamic reticular nucleus (“TRN”; Sherman and Guillery, 2006). There are two classes of inhibitory neurons within TRN: PV neurons and SOM neurons (Clemente-Perez et al., 2017). PV neurons inhibit core relay neurons while SOM neurons inhibit matrix neurons. PV neurons receive input from L6a-CT neurons in the neocortex, while SOM neurons do not receive any input from the neocortex. The axons of all types of thalamocortical relay cells send collaterals to TRN on their way to the neocortex (Clascá et al., 2012). Evidence suggests that these collaterals provide lateral inhibition to nearby relay cells (Pinault and Deschênes, 1998).

## A Model of A Single Macrocolumn

### Layer 4 Stellate Neurons Are Coincidence Detectors on Bottom-Up Input

There is general agreement that layer 4 stellate (“L4-ST”) neurons are the receiver of bottom-up input from lower-order cortical areas, primarily passing information up from the thalamus (Hegdé and Felleman, 2007; George and Hawkins, 2009). Similar to previous models, I propose that L4-ST neurons perform coincidence detection on this bottom-up input (George and Hawkins, 2009). L4-ST neurons provide strong driving input to *all* L2/3 cells within its minicolumn (Douglas and Martin, 2004; George and Hawkins, 2009; Hawkins and Ahmad, 2016). This means that whenever a specific coincidence of input is detected in L4-ST neurons, an entire L2/3 minicolumn will be activated.

Experimental evidence for this simple form of coincidence detection in L4-ST cells can be seen directly in their response properties. Input to L4-ST cells in V1 comes from first-order visual thalamus (LGN), which respond to on-center off-surround circular stimuli in specific locations in their receptive field (Tang et al., 2016). However, L4-ST neurons in V1 primarily respond to bars of light of specific orientations (Martinez and Alonso, 2003). This is exactly what would be expected if L4-ST neurons performed coincidence detection on their bottom-up input. A bar of light in a specific orientation is simply a coincidence of a specific set of on-center, off-surround circles.

### Layer 2/3 Pyramidal Neurons Implement a Competitive Network on Layer 4 Input

The pyramidal neurons found in L2/3 (“L2/3-PY” neurons) have basal dendrites that extend laterally throughout the entire macrocolumn. They have apical dendrites that extend throughout L1 in the macrocolumn. Axonal projections from L2/3-PY neurons project back onto themselves as well as laterally throughout layers 2, 3, and 5 of the entire macrocolumn (Bannister, 2005). L2/3-PY neuron axons synapse on both other L2/3 pyramidal cells as well as inhibitory interneurons that synapse on the soma of nearby L2/3 pyramidal cells (Markram et al., 2004).

I propose the computation of individual L2/3-PY neurons is as described by the “HTM model neuron” in Hawkins and Ahmad (2016): basal dendrites receive “contextual” modulatory input from other L2/3-PY neurons, whereas apical dendrites receive “top-down” modulatory input from other macrocolumns and higher-order thalamus. Excitation of either apical or basal dendrites of L2/3-PY neurons does not provide sufficient depolarization to drive somatic depolarization. However, such subthreshold excitation can modulate the sensitivity of these neurons to L4-ST input, and hence bias the macrocolumn towards different representations (Hawkins and Ahmad, 2016).

When other L2/3-PY neurons synapse directly onto L2/3-PY neuron dendrites, they provide *excitatory* contextual modulatory input. When they instead synapse first onto inhibitory interneurons, they provide *inhibitory* contextual modulatory input. I propose that this excitatory and inhibitory recurrent connectivity enables the L2/3-PY cell network to operate as a winner-take-all competitive network. To illuminate the computational power of such a network, consider the following.

**Figure 4:**
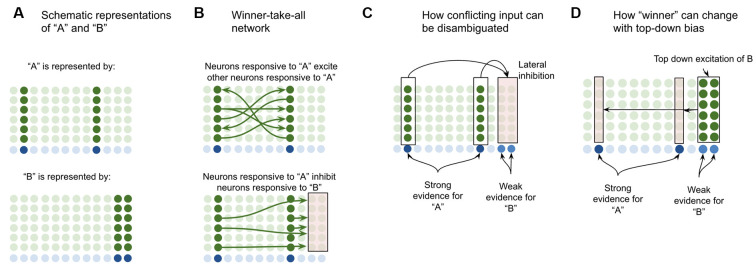
Proposed computation performed by L2/3-PY neurons: winner-take-all network on L4-ST patterns. **(A)** Simplified depiction of how L2/3-PY neurons encode information. A subset of minicolumns are co-activated in response to the coincidence detection of patterns from L4-ST neurons. Example coincident patterns “A” and “B” are shown. For simplicity they are depicted as coincident activity of two minicolumns, in reality patterns may be encoded in more minicolumns. **(B)** Minicolumns that coactivate in response to the same coincident pattern recurrently excite each other (top image in **B**). Minicolumns that do not coactivate in response to the same coincident pattern inhibit each other (bottom image in **B**). This is effectively a winner-take-all network. **(C)** If multiple patterns are detected simultaneously in L4-ST neurons, the winner-take-all network amongst L2/3-PY neurons will ensure that only the coincident pattern with the most evidence (higher L4-ST firing rate) will be activated. **(D)** Top-down bias, implemented through input onto apical dendrites of L2/3-PY neurons, can bias representations towards patterns that have less bottom-up evidence, changing the winner.

Suppose a macrocolumn has learned two coincident patterns in L4-ST neurons—one pattern for “A” and one pattern for “B” (Figure 4A). This model proposes that the L4-ST neurons that respond to “A” will activate a set of minicolumns in L2/3, whereas the different pattern of L4-ST neurons that respond to “B,” will activate a different set of minicolumns in L2/3. I propose that the cells in a minicolumn active within “A” will provide excitatory input to neurons in other minicolumns also active in “A” while providing inhibitory input to neurons in minicolumns that are *not* active during “A” (such as those for “B”). This effectively implements a competitive network, where cells responsive to “A” will excite other cells responsive to “A” while inhibiting those responsive to other stimuli (Figure 4B).

This means that if *ambiguous* or *conflicting* coincidence detection occurs (i.e., both “A” and “B” are input into the network simultaneously), the competitive network in L2/3 will force only one representation to be active (Figure 4C). Furthermore, top-down excitation enables higher cortical regions to bias L2/3 representation, allowing for patterns with less bottom-up input to still win (Figure 4D).

Note that top-down bias cannot create a representation if there is *no bottom-up evidence at all*, it can only bias representations. This is consistent with intuition—consider the famous duck or rabbit example (Supplementary Figure S1). This image can be seen as either a duck or a rabbit, but you can’t see a unicorn. Top-down bias can shift network states between representations that have *some* bottom-up evidence but not to representations with *no* bottom-up evidence.

The proposal here is consistent with many others who similarly propose that L2/3 implements a winner-take-all network (Riesenhuber and Poggio, 1999; Maass, 2000; Yulle and Geiger, 2003; Douglas and Martin, 2004). Consistent with this, recording studies in L2/3 of the visual cortex have shown that neurons selective to different stimuli in the same receptive field seem to laterally inhibit each other, and those responsive to one stimulus are often inhibited during the presentation of other stimuli (Zoccolan et al., 2005; Busse et al., 2009).

### Layer 5a Regular Spiking Neurons Learn and Replay Transitions Between Layer 2/3 Network States

I propose that layer 5a regular spiking (“L5a-RS”) neurons play the computational role of learning and replaying *transitions* between different L2/3 network states. L2/3-PY cells send axonal projections horizontally within L5 (Larsen and Callaway, 2005), providing input to L5a-RS neurons throughout a macrocolumn (Kawaguchi, 2017). L5a-RS neurons send a massive projection back to L2/3 neurons, synapsing both on pyramidal neurons and inhibitory interneurons throughout the macrocolumn (Dantzker and Callaway, 2000; Adesnik and Naka, 2018). This is the perfect circuit set up for the sequential reverberatory activity.

To demonstrate the proposed computation of L5a-RS neurons, let us consider a simplified (but unrealistic) setup where sequences occur in small time windows supportive of Hebbian and spike-timing-dependent plasticity (STDP; this will be generalized later in the article). Suppose you input a rapid sequence of already known patterns (e.g., A, B, and C) into a macrocolumn; and suppose each element follows each other immediately with no delay.

**Figure 5:**
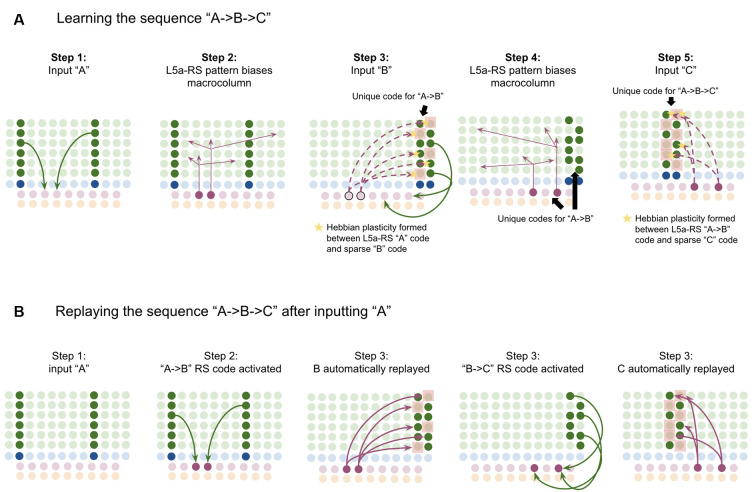
Proposed computation performed by L5a-RS Neurons: learning transitions between L2/3-PY representations. **(A)** Proposed mechanism by which L5a-RS neurons can learn a sequence of L2/3-PY representations. Depicted using simplified representations of “A”, “B”, and “C” (same as in Figure 4). Stars represent synapses where short-term Hebbian potentiation occurs during learning procedure. See text for details. **(B)** Proposed mechanism by which L5a-RS neurons can replay a learned sequence of L2/3-PY representations. See text for details. Red squares denote inhibited neurons. Black outlines denote neurons in subthreshold predictive states.

This model proposes the following learning process will occur. The input of “A” will first activate the minicolumn representation for “A” (step 1 in Figure 5A). This L2/3 pattern for “A” will then activate a pattern separated pattern of L5a-RS neurons (step 2 in Figure 5A). Pattern separation is consistent with observed connectivity—L2/3-PY neurons that are interconnected tend to synapse on L5a-RS neurons that are also interconnected (Kampa et al., 2006). This L5a-RS code is then projected back to the entire L2/3 macrocolumn, where a random biasing of some pyramidal cells will be sub-threshold activated, and some inhibitory interneurons will be sub-threshold activated (step 3 in Figure 5A). I will henceforth refer to this mechanism from L5a-RS neurons as “sequence biasing.” Due to this biasing, when “B” received by the macrocolumn, a more sparser minicolumn representation of “B” will be activated, as opposed to the entire minicolumn. Note that this “sparse” representation of “B” is *unique* to the sequence “A→B.” This is the case because the pattern of neurons inhibited and excited in the minicolumns of “B,” was generated by the L5a-RS neurons specific to “A.” If the preceding element in the sequence were “Z,” then a different sparse pattern of “B” would have been activated. The unique code of “A→B” then similarly activates a pattern separated L5a-RS code (step 4 in Figure 5A). When the pattern “C” is provided, this sequence biasing occurs again—the sparse pattern of “C” that will get activated will be unique to the sequence “A→*B*→C.”

Note that after receiving this sequence once, short term Hebbian plasticity will occur between the L5a-RS code for “A,” and the code for “B” that represents “A→B,” as well as between the L5a-RS code for “A→B” and the code for “C” that represents “A→*B*→C.” Hence now if “A” is input into this macrocolumn, it can automatically replay the entire sequence *via* reverberatory connectivity between L2/3 and L5aRS neurons (see Figure 5B).

However, for the above network to learn the sequence “ABC,” the patterns must be input rapidly within the <100 ms time window for this short-term synaptic potentiation (Markram et al., 1997), which is not realistic. Later in the article, I will generalize this to support realistic timescales.

Experimental data is consistent with the idea that L2/3 representations are sparse and that this sparsity increases over time with learning (Vinje and Gallant, 2002; Yen et al., 2010; Martin and Schröder, 2013). Also, note that I use the term Hebbian plasticity here as interchangeable with STDP. STDP has been shown to be able to learn sequences similarly to how I describe above, providing support for the plausibility of the proposed learning mechanism (Brea et al., 2012, 2013; Rezende and Gerstner, 2014; Osogami and Otsuka, 2015). Further, STDP has also been shown specifically within L2/3 synapses (Froemke et al., 2005; Bender et al., 2006; Nevian and Sakmann, 2006).

### Layer 5b Intrinsically Bursting Neurons Perform Pattern Separation on Layer 2/3 Output to Generate Unique “Sequence Codes”

Axons of layer 5b intrinsically bursting (“L5b-IB”) neurons represent the key output code of the macrocolumn: L5-IB neurons project directly to motor areas, striatum, and provide driving input to higher-order thalamic relay neurons that project to higher-order cortical areas (Kim et al., 2015; Baker et al., 2018).

**Figure 6:**
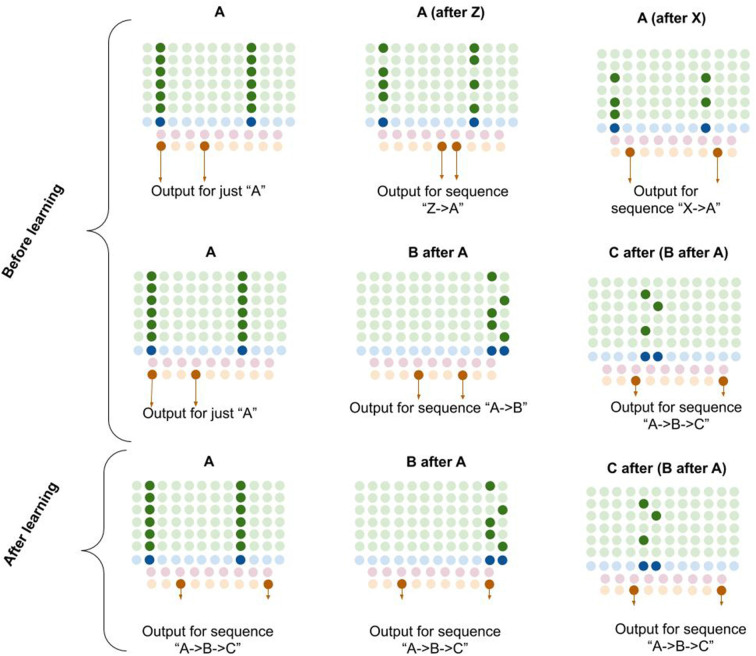
Proposed computation performed by L5b-IB neurons: generation of unique sequence codes. I propose that before learning, L5b-IB neurons perform pattern separation on L2/3-PY representations (top two rows). After learning, I propose L5b-IB neurons cluster together different representations into a common sequence code (bottom row). See text for details.

Computationally, I propose that L5b-IB neurons perform pattern separation on the L2/3 macrocolumn code, meaning that the L5b-IB code is sensitive to the *sequence* representation in L2/3, not just the column representation (see Figure 6). I propose that this “unique sequence code” is the core output code of a macrocolumn.

The observed connectivity is consistent with such pattern separation. L2/3-PY neurons project horizontally *within* L5 of a macrocolumn (Larsen and Callaway, 2005) making dense connections with both L5b-IB neurons as well as L5 inhibitory interneurons (Thomson and Bannister, 2003; Kawaguchi, 2017). L5b-IB neurons also have reciprocal horizontal connectivity with each other (Naka and Adesnik, 2016). L2/3-PY neurons that are reciprocally connected tend to synapse onto L5b-IB neurons that are also reciprocally connected (Kampa et al., 2006).

In Figure 6, you can see visually how L5b-IB output will be radically different based on the sequence code represented in L2/3-PY. The *same* current pattern representation (“A”) will have very different output codes depending on the *prior* elements in the sequence. In other words, the output code for “A” coming right after “Z” is completely different from the output code for “A” coming right after “X.”

Furthermore, if you chain elements together, the L2/3 representation in the *final* element of the sequence triggers an L5-IB output representation that is unique for that exact sequence. So in bottom right example in Figure 6, you can see that the columnar representation of “C” encodes “A→*B*→C” (from dynamics described in L5a-RS neurons), and hence the L5b-IB output is a unique code representing the exact sequence “A→*B*→C.” This provides a mechanism for how a macrocolumn can output a unique sequence code based on its inputs.

I propose that after learning, the L5b-IB sequence code will stabilize across the whole sequence to represent the pattern of the last element. Later in this article, I will describe in detail how this occurs.

Although far from conclusive, evidence of L5b-IB response properties is at least consistent with the proposal that they generate invariant sequence codes. First, L5b-IB neurons have been shown to have much wider receptive fields than L5a-RS or L2/3-PY neurons (Sun et al., 2013), which is consistent with the idea that they cluster groups of commonly occurring sequences of elements in L2/3 together into a stable sequence code. Second, in visual cortex L5b-IB neurons tend to be “complex cells” (Gilbert, 1977), responsive to complex patterns of input, consistent with the proposal that L5b-IB represents further processed information after L4-ST coincident patterns.

### Layer 6a Corticothalamic Neurons Make Top-Down Predictions of Next Elements in Sequences

I propose that layer 6a corticothalamic (“L6a-CT”) neurons encode *predictions* of the upcoming stimuli a macrocolumn expects. The observed connectivity of L6a-CT neurons is consistent with this. L6a-CT neurons have apical dendrites in L4, where they have access to direct input from core thalamic neurons (Thomson, 2010). Dendritic NMDA spikes in these apical dendrites can learn the same coincidences that L4-ST dendrites do. Consistent with the idea that L6a-CT neurons learn similar coincidences to L4-ST neurons, the response properties of L6a-CT neurons in the visual cortex are of the “simple” type, responding to bars of specific orientations just like L4-ST neurons do (Hirsch et al., 1998). L6a-CT neurons receive driving input from L5B (Zarrinpar and Callaway, 2006), which I speculate comes from L5B-IB neurons. L6a-CT neurons project both to L4-ST neurons as well as interneurons within layer 4 that inhibit L4-ST neurons (Thomson, 2010; Kim et al., 2014). The majority of excitatory input to L4-ST neurons does not come from the thalamus, but rather from L6a-CT neurons (Ahmed et al., 1994; Binzegger et al., 2004). These projections seem to be modulatory and not driving (Kim et al., 2014). I hypothesize that these L4 projections provide subthreshold excitation of L4-ST neurons predicted to become active, and inhibition of L4-ST neurons predicted to *not* become active. Consistent with this, photostimulation of L6a-CT neurons is inhibitory of most L4-ST neurons while modulating the “gain” of their responses to their preferred stimuli (Olsen et al., 2012; Kim et al., 2014), which is exactly what you would expect if L6a-CT neurons are making specific predictions of upcoming sensory input.

**Figure 7:**
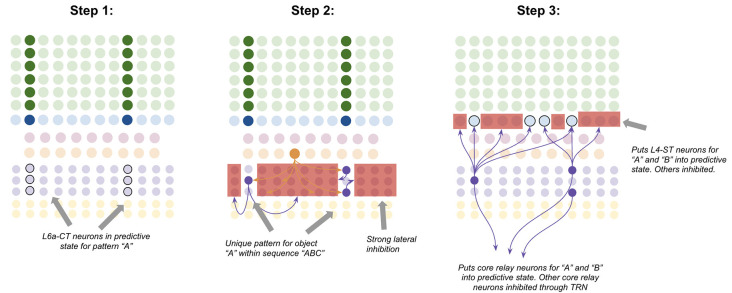
Proposed computation performed by L6a-CT neurons: predicting next element In A sequence. Step 1: bottom-up input from lower-order thalamus puts a subset of L6a-CT neurons into a predictive state, representing the same coincident pattern encoded in L4-ST neurons (e.g., “A”). Step 2: L5b-IB sequence code activation for the predicted sequence (e.g., “ABC”) activates a random subset of L6a-CT neurons. Due to extensive lateral inhibition amongst L6a-CT neurons, only the neurons that were in a predictive state end up firing action potentials. This produces a sparse pattern in L6a-CT neurons that is unique to the element “A” within the sequence “ABC.” Step 3: L6a-CT neurons that encode the element “A” within sequence “ABC” predict the elements “A” or “B” as upcoming elements in sequence. This L6a-CT pattern subthreshold activates (“predictive state”) core thalamic relay neurons and L4-ST neurons responsive to “A” and “B” and inhibits core thalamic relay neurons and L4-ST neurons not responsive to “A” or “B”. See text for more details. Red squares denote inhibited neurons. Black outlines denote neurons in subthreshold predictive states.

L6a-CT neurons have very little recurrent connectivity, but have substantial lateral inhibition of each other, similar to L2/3-PY neurons (Thomson, 2010). This means that the winner-take-all dynamic proposed to occur in L2/3, can also occur in L6a-CT, albeit with different computational consequences due to a different input. Consider the following—suppose a specific coincident pattern of input is received by a macrocolumn. This puts a specific pattern of L4-ST neurons into an *active* state, as well as putting a specific pattern of L6a-CT neurons into a *predictive* state (Figure 7, step 1). Furthermore, suppose a given L5b-IB sequence code sends driving input to a random subset of L6a-CT neurons. When the L5b-IB sequence code fires, only the predicted L6a-CT neurons receiving L5b-IB input will become active, the rest will be inactivated by lateral inhibition. This generates a sparse L6a-CT code that is unique to a specific element within a specific sequence (Figure 7, step 2). In other words, the “B” in “ABC” will trigger a different L6a-CT code than the “B” in “DBF.” This enables the L6a-CT projection to predict the next element in the sequence based on the current element as well as the sequence it is in. In other words, if receiving “B” in “ABC,” L6a-CT will predict “C,” if receiving “B” in “DBF,” L6a-CT neurons will predict “F.”

Given the above, it is not hard to imagine how learning these associations might occur. The random L6a-CT pattern that gets activated by “B” in the sequence “ABC” will fire right before the core thalamic neurons and L4-ST neurons for “C,” hence building short-term Hebbian plasticity with both of these neurons. Hence if the sequence “ABC” is replayed a sufficient quantity of times, these sparse L6a-CT codes will build long-term plasticity with the core thalamic and L4-ST neurons that tend to follow them, hence reliably predicting the upcoming element in a learned sequence.

## Frontal Input—Motor Commands, Attention, and Working Memory

The above model of a single macrocolumn can be used to explain the neural mechanisms of various cognitive functions of the sensory cortex. It has been shown that the frontal cortex sends extensive projections directly to the basal dendrites of L6a-CT neurons and the apical dendrites of L2/3-PY neurons (Nelson et al., 2013; Leinweber et al., 2017). Under the assumption that macrocolumns function as proposed in the above model, I will show that this projection can explain the neural mechanisms for motor prediction, attention, and working memory.

### Motor Predictions: Layer 6 Corticocortical and Layer 6a Corticothalamic Neurons Integrate Motor Commands to Predict Upcoming Sensory Input

I propose that one function of these frontal projections to L6 in the sensory cortex is to enable L6a-CT neurons to incorporate volitional motor commands into their prediction of upcoming sensory input. To see how macrocolumns might accomplish this, consider an example of a saccadic eye movement.

**Figure 8:**
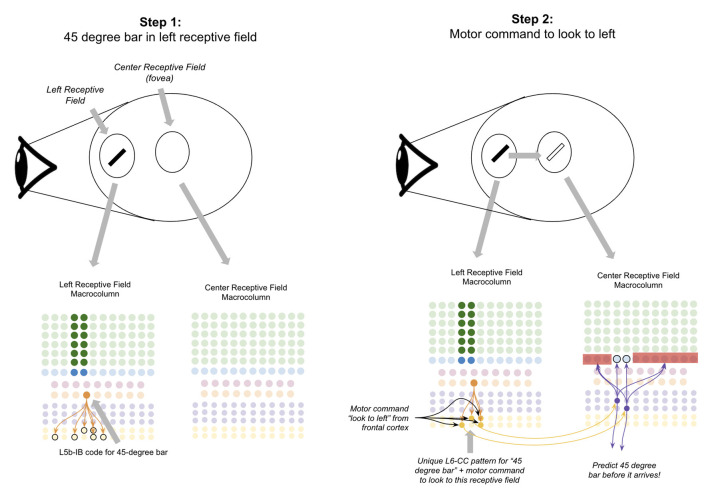
Proposed computation performed by L6-CC neurons: integrating motor commands into predictions of upcoming sensory input. Step 1: the top oval to the right of the “eye” depicts a subject’s retina where a 45-degree black bar exists in their left receptive field, and nothing exists in the subject’s fovea (center receptive field). The macrocolumns processing information from two receptive fields (shown in circles) are depicted below the graphic of the retina. Step 2: a volitional motor command to “look towards the left” will project from frontal cortex to L6-CC neurons, which project out a unique pattern for “45-degree bar” plus “motor command to look to left.” After learning, L6a-CT neurons within the center receptive field will respond to this pattern and thereby predict the upcoming 45-degree bar in the center receptive field before it arrives. Red squares denote inhibited neurons. Black outlines denote neurons in subthreshold predictive states. See text for more details.

Suppose there is a 45-degree bar in your left visual field, and you decide to move your eyes to look at it (Figure 8, step 1). If this network works as proposed, the L6a-CT neurons in the fovea of your visual field should predict the 45-degree bar before it occurs. To accomplish this, the macrocolumn(s) processing your left receptive field must somehow “transfer” the representation of the 45-degree bar to the macrocolumn(s) processing your fovea receptive field *before* you look to the left.

I propose the mechanism for how this occurs is through long-range projections from L6-CC neurons. L6-CC neurons send projections from their macrocolumn to far away macrocolumns within the same level of their hierarchy (Harris and Mrsic-Flogel, 2013). These L6-CC neurons synapse onto other L6-CC and L6a-CT neurons both in distant macrocolumns as well as within their own macrocolumn (Bremaud et al., 2007). L6-CC neurons also receive substantial input from L5B, which I speculate comes from L5B-IB neurons (Zarrinpar and Callaway, 2006). I propose that by integrating the input from frontal motor commands and local sensory stimuli, L6-CC neurons respond to specific coincidences of a motor command and sensory stimulus. In our example, the “look to the left” motor command along with the 45-degree bar will activate a specific pattern of L6-CC neurons (Figure 8, step 2). This pattern of L6-CC neurons will send long-range projections to the L6a-CT neurons in the fovea macrocolumn to trigger the prediction of a 45-degree bar before it occurs (Figure 8, step 2). Learning this mapping can occur with simple STDP—whenever you have a 45-degree bar in your left visual field and you look to the left, you will always end up with a 45-degree bar in your fovea. As such, this mapping will be built naturally with a sufficient amount of visual exploration. This proposal is consistent with the observation that deep layer neurons within the sensory cortex exhibit movement-related response properties *before* movement begins, even without any changes in sensory input (Jordan and Keller, 2020).

Doing this representational transfer with comprehensive macrocolumn-to-macrocolumn connectivity would likely lead to a combinatorial explosion—each macrocolumn would have to learn a mapping between an object in every other macrocolumn and a given saccadic motor command. However, there are several shortcuts one could imagine that would make this more feasible. First, matching could be made fuzzily, which is intuitive as object recognition outside of the fovea is already dramatically reduced. Second, matching could be made to groups of stimuli instead of only one (e.g., all bars between 0 and 45 degrees in left visual field maps to all bars between 0 degrees and 45 degrees in the fovea). Third, connectivity could be highly biased from peripheral visual field macrocolumns to fovea field macrocolumns and have very little transference from the fovea to peripheral fields. All of these would dramatically reduce the required connectivity of L6-CC neurons while still enabling the overarching motor prediction mechanism to function.

### Top-Down Attention: Frontal Projections to L6a-CT and L2/3-PY Neurons Enable Attention

I propose that another function of the frontal projection to L6a-CT and L2/3-PY neurons in the sensory cortex is to enable top-down attention. I use the term “top-down attention” to refer to two abilities (Knudsen, 2007)—the ability of a subject to toggle between different possible interpretations of ambiguous stimuli (“duck or rabbit?” see Supplementary Figure S1) and the ability of a subject to search an environment for specific features or objects (e.g., “where’s waldo?”). If macrocolumns work as proposed here, then frontal input to apical dendrites of L2/3-PY neurons will bias representations and hence can disambiguate stimuli the same way we already proposed that higher-order sensory macrocolumns do. Further, when searching for a specific stimulus or object, frontal input to the basal dendrites of L6a-CT neurons will put neurons selective for certain features or objects (e.g., “waldo”) into a predictive state the same way motor projections and bottom-up sensory input puts L6a-CT neurons into predictive states. This “prediction” of a sensory stimulus will make the network much more responsive to the predicted features within an environment, enabling rapid recognition when receiving consistent stimuli (“aha! There is waldo!”).

The idea that top-down attention works by biasing representations in the L2/3 winner-take-all network and making predictions through L6a-CT neurons is consistent with a broad set of experimental evidence. It has been shown that top-down attention of a specific feature or object increases the responsiveness of specifically the neocortical neurons that are tuned to that feature or object (Moran and Desimone, 1985; Desimone and Duncan, 1995; Maunsell and Treue, 2006), while simultaneously *decreasing* the responsiveness of neurons that are tuned to other feature or objects (Chelazzi et al., 1993; Treue and Trujillo, 1999; Vanduffel et al., 2000; Reynolds and Desimone, 2003). This has been replicated repeatedly across different modalities and hierarchical levels of sensory cortex (Motter, 1993; Treue and Maunsell, 1996; Luck et al., 1997; Chelazzi et al., 1998; Reynolds et al., 1999; Recanzone and Wurtz, 2000; Chelazzi et al., 2001; Kastner and Ungerleider, 2001; McAdams and Reid, 2005). Further, it has been shown that the higher the attentional demand in the task, the greater the increase in the sensitivity of the neurons being attended to Williford and Maunsell (2006) and Martínez-Trujillo Julio and Treue (2002). The broad idea that attention is fundamentally a process of biased competition in a winner-take-all network is consistent with prior models of attention (Lee et al., 1999; for reviews see Reynolds and Chelazzi, 2004; Knudsen, 2007). There is also evidence that attention modulates the responses of TRN (McAlonan et al., 2006), as would be expected by this model.

### Working Memory: Frontal Projections to L6a-CT Neurons Can Trigger and Maintain Specific Memories

A recent experimental study showed that L6a-CT neurons provide strong driving input to L5a-RS neurons, eliciting action potentials directly (Kim et al., 2014). If macrocolumns work as proposed here, then the activated L5a-RS neurons will activate L2/3-PY neurons. This means that if the frontal cortex triggers a specific L6a-CT representation, then simultaneously it will trigger a corresponding L2/3-PY representation *via* L5a-RS neurons. In other words, a frontal projection to L6a-CT can trigger and maintain L2/3-PY representations without sensory input.

There is reasonable evidence that working memory operates this way. It has been shown that sensory cortex shows delay activity during working memory tasks and that this activity is specific to deep and superficial layers, avoiding L4, exactly what would be predicted by the above scheme if L6a-CT neurons trigger representations in L2/3-PY bypassing L4 (Lawrence et al., 2018). It has been shown that maintaining specific features in working memory selectively activates neurons selective to those features in the sensory cortex (Harrison and Tong, 2009; Serences et al., 2009; Tong, 2013). It has been shown that top-down projections from higher-order cortex during delay periods project specifically to deep layers (Miyashita, 2019). Further, it has been shown that during delay periods, firing starts in infragranular layers of sensory cortex and then propagates to superficial layers, while during sensory experiences processing starts in granular layers, propagates to L2/3, and then propagates to deeper layers (Sakata and Harris, 2009; Takeuchi et al., 2011).

I propose that the hippocampus is an essential component of this process. CA1 within the hippocampus has been shown to replay place codes on the gamma rhythm during working memory tasks (Chadwick et al., 2015; Drieu and Zugaro, 2019). CA1 of the hippocampus provides an extensive excitatory projection to the frontal cortex (Jay et al., 1989, 1992; Jay and Witter, 1991; Carr and Sesack, 1996; Tierney et al., 2004; Hoover and Vertes, 2007). If CA1 triggers replay in the frontal cortex, then the corresponding representations within the sensory cortex could also be replayed due to already described frontal projection to L6a-CT neurons. Consistent with this, it has been shown that neural activity between the hippocampus and neocortex are correlated during working memory tasks and that frontal firing lags behind the hippocampal firing, suggesting information flows from hippocampus to frontal cortex (Hyman et al., 2005, 2010; Jones and Wilson, 2005; Siapas et al., 2005; Benchenane et al., 2010; Sigurdsson et al., 2010). L6 in higher-order sensory cortex also receives a direct projection from CA1, providing another more direct mechanism by which this the hippocampus may trigger memories in the absence of sensory input (Cenquizca and Swanson, 2007).

## Thalamocortical Networks Coordinate Processing Using Oscillations

Let us now turn to answer the question of how the brain coordinates processing across macrocolumns on precise timescales. Processing on precise time scales is an essential requirement for networks of macrocolumns. Postsynaptic excitation after presynaptic excitation across a single synapse, in the absence of successfully driving a postsynaptic spike, typically decays for 10–30 ms (Curtis and Eccles, 1959; Sayer et al., 1990; Williams and Stuart, 2000). This means that in order for dendritic segments to sum inputs across multiple synapses, presynaptic neurons must fire action potentials within a precise time window.

### Macrocolumns Oscillate Between “Input States” and “Output States”

I propose processing on precise timescales is made possible by macrocolumns oscillating back and forth between an “input state” and an “output state.” The inherent circuit dynamics within the thalamus ensure that macrocolumns oscillate between these states at the same time, enabling coordinated processing. Within the thalamus, about ~30% of thalamocortical cells have been called “High-Threshold Busting Cell” (HTC) due to their rhythmic bursting at the alpha rhythm (Lörincz et al., 2009; Hughes et al., 2011). When these HTC neurons burst fire they inhibit other thalamic relay neurons *via* thalamic interneurons (Lörincz et al., 2009). I speculate that these HTC cells are in fact the same as the multiareal matrix cells identified by Clascá et al. (2012), and the neurons they inhibit are core relay neurons. If this is true, then on the alpha rhythm, multiareal matrix neurons will fire for ~50 ms while core neurons pause, and then core neurons will fire for 50 ms while multiareal matrix neurons pause, back and forth.

**Figure 9:**
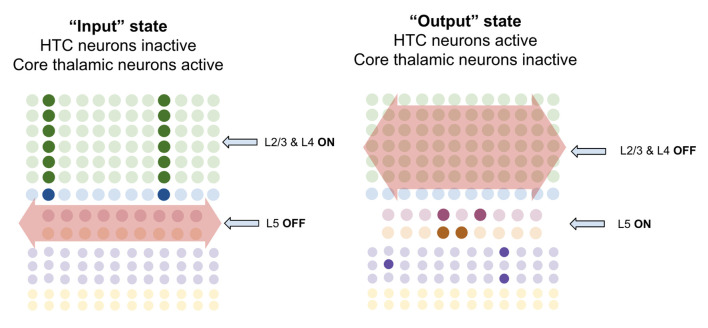
Macrocolumns are proposed to always exist in either an “input state” or an “output state.” Input states are proposed to occur when high-threshold bursting cells in thalamus pause. During input state superficial layers (L2/3 and L4) are active while deep layers (L5/6) are inhibited. Output states are proposed to occur when high-threshold bursting cells in thalamus burst fire. During output states superficial layers (L2/3 and L4) are inactivated while deep layers (L5/6) are activated. See text for details.

I propose that when multiareal neurons pause and core thalamic neurons are activated, macrocolumns lock into an “input state.” In this state, macrocolumns integrate bottom-up input from core thalamic neurons through L4-ST neurons and top-down input through apical dendrites of L2/3-PY neurons. Activation of L4-ST neurons excites inhibitory interneurons in L5 which directly inhibit L5a-RS and L5b-IB neurons (Pluta et al., 2015; Naka and Adesnik, 2016). Hence during input states, superficial layers are activated, and deep layers are inactivated (Figure 9).

However, when multiareal matrix neurons burst fire and core relay neurons pause, the macrocolumn shifts to an “output state.” In this state, I propose that L2/3-PY and L4-ST neurons will be inhibited, while deep layer neurons will become activated. There are several ways in which this could happen. Multiareal matrix cells project to L1 (Vijayan and Kopell, 2012), where thalamocortical neurons synapse onto thick apical dendrites of L5b-IB neurons (LaBerge, 2005) driving burst-firing. L5b-IB neurons then synapse onto L6a-CT neurons (Thomson, 2010) which activate L6 interneurons that inhibit layers 4 and 2/3 (Bortone et al., 2014). This mechanism is consistent with the observation that L5b-IB neuron firing triggers up/down states within L2/3 by propagating first to L6 and then to superficial layers (Lórincz et al., 2015). Another mechanism could be through multiareal matrix neurons directly projecting to inhibitory interneurons in layer 2/3 that inhibit L2/3-PY and L4-ST neurons (Vijayan and Kopell, 2012). This direct inhibition is consistent with the observed connectivity of multiareal matrix neurons to layer 2/3 of the higher-order cortex. Furthermore, thalamic projections within L1 also synapse onto inhibitory interneurons which inhibit L2/3 neurons (Cruikshank et al., 2012), providing a mechanism by which multiareal matrix neurons may inhibit top-down input onto L2/3-PY neurons.

I propose there are three key computational purposes of this output state: First, the output state enables a stable output of the L5b-IB sequence code, so that it can be passed to other regions without being interrupted by changes in sensory input. Second, the output state enables the macrocolumn to reactivate memories within L2/3-PY *via* L6a-CT neurons without being disrupted by incoming sensory information through L4-ST. Third, it provides a mechanism for macrocolumns to “reset” their representations in concert, and hence enable a network to re-lock into a new representation given new information.

### Network Oscillations for Integrated Processing: Passive Processing at Alpha and Attentive Processing at Theta

I propose that there are two broad oscillatory modes of sensory thalamocortical networks: passive processing and attentive processing, each coordinating processing between different sets of regions at different frequencies.

I propose passive processing is the default thalamocortical network mode within the sensory cortex. In passive processing macrocolumns oscillate between input and output states at the alpha rhythm, spending roughly ~50 ms in each state. These alpha oscillations are driven by the inherent oscillatory dynamics of HTC cells and L5b-IB burst firing as described in the prior section.

However, I propose that during situations requiring top-down attention or working memory, thalamocortical networks slow down their oscillations to the theta frequency (~100 ms in each state). I propose that the purpose of this oscillatory slowing is threefold. First, the default oscillatory dynamics of the higher-order frontal cortex and hippocampus are in the theta frequency (Buzsáki, 2002; Colgin, 2011), hence to coordinate processing with those regions’ sensory cortex needs to also oscillate at the same rhythm. Second, this slowing down gives L2/3-PY neurons *more time* in between input states to replay sequences, hence enabling more items to be stored in working memory. Third, this slowing gives L2/3-PY neurons more time to lock into a representation that well matches top-down input and bottom-up input. I proposed that during periods of a good match between top-down expectations and bottoms up input, L2/3-PY neurons resonate at gamma oscillations. This is consistent with the observations of strong gamma oscillations within L2/3 during attention (Buffalo et al., 2011), as well as the observed entrainment of gamma to theta oscillations (Soltesz and Deschênes, 1993; Bragin et al., 1995; Lee et al., 2005; Canolty et al., 2006; Colgin et al., 2009; Belluscio et al., 2012). As proposed by others, I hypothesize that the function of these rapid oscillations during successful predictions facilitates long-term synaptic plasticity to learn new associations of objects and sequences being attended to Grossberg and Versace (2008).

There are several mechanisms by which oscillations in the sensory cortex might be slowed from alpha to theta during attentive processing. It has been shown that L5b-IB neurons can modulate their bursting rate within ranges encapsulating both theta and alpha frequencies based on apical input (Li et al., 2013). It is possible that independent pacemakers in the septal complex become independently activated during attentive states (Petsche et al., 1962). CA1 from the hippocampus to the higher-order sensory cortex may modulate oscillations during attention. The frontal cortex also sends a large projection to the sensory cortex through the claustrum, which may trigger or modulate oscillatory states (Narikiyo et al., 2020; White et al., 2018). It is also possible that various arousal neuropeptides or neuromodulators change inherent oscillatory dynamics in the thalamus and cortex (Li et al., 2017).

### Unraveling the Experimental Data on Oscillations

The proposal here is definitively not a comprehensive account of all neural oscillations. However, the theory presented here well accounts for a large body of findings regarding specifically theta and alpha oscillations.

The alpha frequency is the strongest EEG oscillatory signal observed in the brain of awake subjects (Berger, 1929; da Silva et al., 1973; Lopes da Silva and Niedermeyer, 1999). Further, these studies showed that alpha activity is greatest when humans are awake, but not engaged in any specific task. Both consistent with the idea that alpha oscillations are a form of “passive” processing.

There is also experimental evidence suggesting that theta oscillations are triggered specifically under conditions of high attention. Local theta rhythms are observed when engaging in selective attention, specifically in the modality being attended to (Green et al., 2011). Theta oscillations have been observed when animals are navigating spatial environments, a task presumably required substantial attention (Caplan et al., 2003; Tsanov et al., 2011). During working memory tasks, there is sustained theta activity within the neocortex during the delay period (Gevins et al., 1997; Raghavachari et al., 2001; Jensen and Tesche, 2002; Scheeringa et al., 2009).

There is also evidence that not only does theta increase under attentive tasks, but alpha decreases specifically in the modality being focused on, consistent with the idea that networks shift from alpha oscillations to theta oscillations. It has been shown that when focusing on motor tasks, there is an increase in alpha over visual areas, and when focusing on visual tasks, and increase in alpha over motor areas (Pfurtscheller, 1992). When focusing spatial attention to one side, alpha increases over the side of the brain not processing the attended location, whereas alpha decreases over the side that is processing the attended location (Worden et al., 2000; Thut et al., 2006; Rihs et al., 2007; van Gerven and Jensen, 2009; Kelly et al., 2009; Haegens et al., 2010; Händel et al., 2011).

This proposal is also consistent with the observed laminar origins of various oscillations, where spiking activity within superficial layers is most coherent with gamma, activity in deep layers is most coherent with alpha (Livingstone, 1996; Buffalo et al., 2011), and gamma oscillations in superficial layers are entrained to alpha oscillations in deep layers (Jensen and Mazaheri, 2010).

There is admittedly experimental evidence that is inconsistent with the proposal herein. Most notably, some studies have shown that attention actually decreases theta power (Spyropoulos et al., 2018). I hypothesize that this inconsistency arises due to the unreliability of using changes in the relative power of different frequencies observed in local field potentials to ascertain underlying oscillatory processes. For example, if under a moderate level of attention input states last for 100 ms, and output state last for 100 ms, but then under more strenuous attention input states prolong themselves to 150 ms, and the output state only lasts for 50 ms, this would be observed in Fourier analysis as a decrease in theta power. However, in this latter case, the actual theta oscillatory process did not become weaker, rather macrocolumns simply modulated their times within input and output states to prolong integration time.

There is also experimental evidence that alpha oscillations primarily pass information in the feedback direction, while theta and gamma oscillations pass information in the feedforward direction (Kerkoerle et al., 2014; Bastos et al., 2015; Spyropoulos et al., 2018), a phenomenon not directly explained by this theory. Although speculative, it is possible to explain these findings in the context of this theory under the following assumption. If it were the case that the mechanism by which frontal cortex transitions sensory cortex from passive processing to attentive processing occurs first in the *lower*-order cortex, whereas the frontal disengagement that transfers networks back from attentive processing to passive processing occurs first in the *higher-order* cortex, then this theory can explain these findings. In such a case, attentive states would always occur first in the lower-order cortex and propagate upwards, and passive states would always occur first in the higher-order cortex and propagate downwards, hence showing the observed differential in directions of frequency propagation.

However, it is important to note that there are several alternative interpretations of neural oscillations that are also consistent with experimental data, and are more consistent with alternative models of the neocortical microcircuit (Wang, 2010; Bastos et al., 2012; Doesburg et al., 2015). Further work will have to be done to unify and/or disambiguate these interpretations.

## How Networks of Macrocolumns Recognize Already Learned Objects and Sequences

### The Computational Function of Networks of Macrocolumns

With the above work done, the next question is: what are the emergent computations of hierarchical thalamocortical networks of these macrocolumns? I propose these hierarchical networks of macrocolumns serve two purposes: (1) “integration”—progressively more stable representations of input get formed higher in the hierarchy, as proposed by HTM theory (George and Hawkins, 2009); and (2) “disambiguation”—conflicting patterns are disambiguated higher in the hierarchy, and this is used to bias patterns in macrocolumns lower in the hierarchy.

The integration enables broad inputs across thousands of macrocolumns to be represented in fewer macrocolumns over several levels of a hierarchy until an L2/3 representation in a higher-level macrocolumn could represent a coincidence pattern over thousands of lower-level macrocolumns. Sequence outputs of one level of macrocolumns become coincident objects within L2/3 representations of the level above. Taken together, higher-level L2/3 representations come to represent sequences of sequences of sequences.

Disambiguation can occur in two specific ways: (a) sequence disambiguation—an ambiguous sequence can be disambiguated (e.g., macrocolumn gets “A,” but can’t tell the difference between two sequences “ABC” and “AZY”) and (b) object disambiguation—an ambiguous input can be disambiguated (e.g., macrocolumn gets “A” and “B” simultaneously, which input is right?).

### Sequence Disambiguation

**Figure 10:**
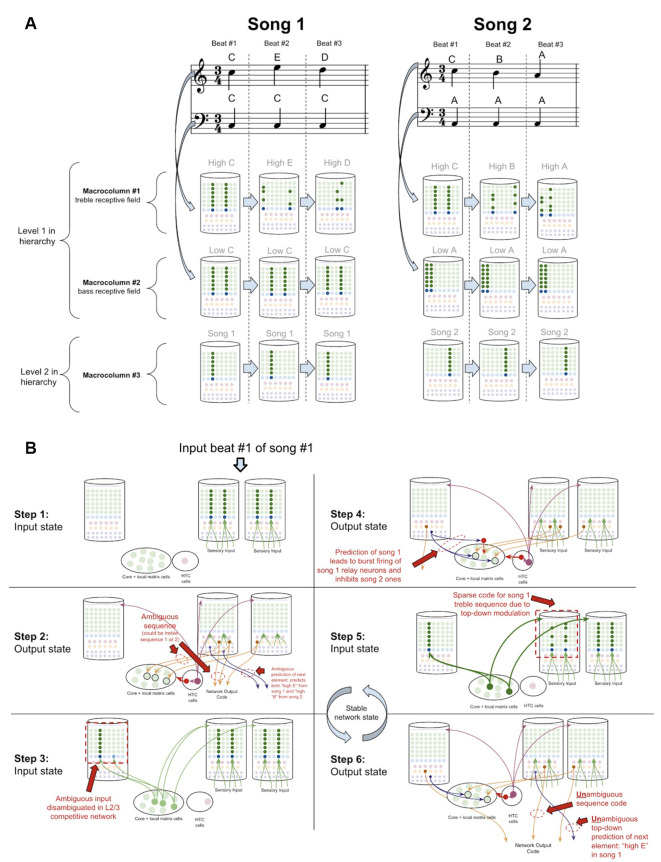
How networks of macrocolumns may perform sequence disambiguation. **(A)** Depiction of how two songs might be depicted in A 2-Level network of three macrocolumns. Example song 1 and song 2 are shown in musical notation, each composed of only three beats. Macrocolumn #1 only receives input from “high notes” in the treble clef (top notes), and macrocolumn #2 only receive input from “low notes” in the bass clef (bottom notes). Macrocolumn #3 receives input only from macrocolumns #1 and #2. This architecture is consistent with observed neuroanatomy—it is known that auditory cortex is organized tonotopically (see text). The L4-ST and L2/3-PY representations active on each beat in each song are depicted below each beat. Note that macrocolumn #3 has two different representations of song 1 and song 2 and can disambiguate which song is being played even on beat #1. **(B)** Using the model of the two songs in **(A)**, this figure shows the step by step process by which macrocolumn #1 can disambiguate between the two songs on beat #1 using top—down feedback. All macrocolumns in this network are oscillating between “input states” and “output states” at the same time—each step depicted represents an oscillatory phase of the network. See text for details.

To demonstrate how the sequence disambiguation occurs, let us consider how a simple network of macrocolumns could learn to differentiate between two songs (Figure 10A). Consider a two-layer network of three macrocolumns. Suppose there are two “level-1” macrocolumns, each of which receives auditory input within a certain frequency band, as is seen in the tonotopic mapping of the auditory cortex (Saenz and Langers, 2014). Let us say macrocolumn #1 receives only “high notes” (treble) input, and macrocolumn #2 receives only “low notes” (bass) input. And let us say the output L5b-IB codes from both macrocolumn #1 and #2 are passed through the thalamic relay to L4-ST neurons in macrocolumn #3 (hence implementing a two-level hierarchy).

Suppose this network has only ever heard one of two songs (song 1 and song 2 in Figure 10A). In song 1, the treble range plays C, E, D, and the bass just plays C. In song 2, the treble plays C, B, A, and the bass just plays A. Hence macrocolumn #1 knows two sequences: C→*E*→D, and C→*B*→A. This means that on beat #1, macrocolumn #1 *does not know* what song is being played; it is within an ambiguous sequence.

However, any human that heard these two songs just once, would *immediately* be able to predict the next treble note that would be played on beat #2 after hearing beat #1. It is clear that the network has sufficient information to disambiguate which song is being played: if the bass note is C, then we know we are in song 1 and the beat #2 treble note is E, on the other hand, if the bass note is A, then we know we are in song 2 and the beat #2 treble note is B.

To see how this network of macrocolumns implements this sequence disambiguation, let us play out the processing steps in our two-level network of macrocolumns (see Figure 10B). Step 1 begins when beat #1 notes are played (“high C” and “low C”). During the input state, macrocolumn #1 locks into a representation for “high C,” and macrocolumn #2 locks into a representation for “low C.” In the output state (step 2), macrocolumn #1 will activate *two* *different* learned sequence code outputs, since it is ambiguous whether the sequence “CED” or “CBA” will be played. For simplicity, I depict only a single L5b-IB neuron firing for a given sequence representation. These sequence outputs from L5b-IB neurons in level 1 macrocolumns activate specific relay neurons in the higher-order thalamus and then provide input to L4-ST of macrocolumn #3. The coincidence detection in the L4-ST neurons of macrocolumn #3 now receives conflicting evidence from these lower-level macrocolumns. Two relay neurons are consistent with song 1, and one relay neuron is consistent with song 2. Given the competitive network in L2/3, the representation with the most evidence (i.e., song 1) will win out. Hence the representation for only song 1 gets activated in L2/3 (step 3).

Note, the ability to co-activate multiple representations in L5b-IB, without a winner-take-all mechanism preventing such co-activation, is in stark contrast to the L2/3 network and is consistent with the experimental data on L5b-IB neurons. The inhibition observed amongst L5b-IB neurons seems to not implement lateral inhibition, but rather drive coordinated burst firing amongst L5b-IB neurons. The L5b-IB to L5b-IB inhibition exhibits a remarkable delay in firing, inhibiting other L5b-IB neurons only after 100–200 ms (Silberberg and Markram, 2007). This is too slow to implement a winner-take-all mechanism—alternative representations have a long-time window to be co-active together. Further, L5b-IB inhibition is not selective for only other L5b-IB but also provides feedback inhibition back onto themselves (Naka and Adesnik, 2016), supportive of the idea that the role of this inhibition is to coordinate burst firing and not to implement lateral inhibition.

In the next output state of the network (step 4), an unambiguous sequence code gets output from macrocolumn #3. Furthermore, the L6a-CT neuron back-propagation provides modulatory input back to the thalamic relay neurons for song 1, while* inhibiting* relay neurons representing song 2. Hence, in the next input state (step 5), there will be only excitement of thalamic relay neurons active during song 1, which thereby provides biased top-down input to macrocolumn #2. This top-down bias leads to the activation of the L2/3 representation of only the C within the sequence CED, and not the C within sequence CBA. In the next output state (step 6), macrocolumn #2 will now output an unambiguous sequence code only for the sequence CED. Further, this will lead to an unambiguous prediction of “high E” as the next element in the sequence through L6a-CT neurons in macrocolumn #2. At this point, this network has now achieved a stable state, and if sensory input is unchanged, this network will oscillate back and forth between step 5 and step 6.

This sequence disambiguation could happen within the timescale of hundreds of milliseconds, and it can enable this network to unambiguously know that we are playing song 1 and not song 2, even when just hearing beat #1.

### Object Disambiguation

**Figure 11:**
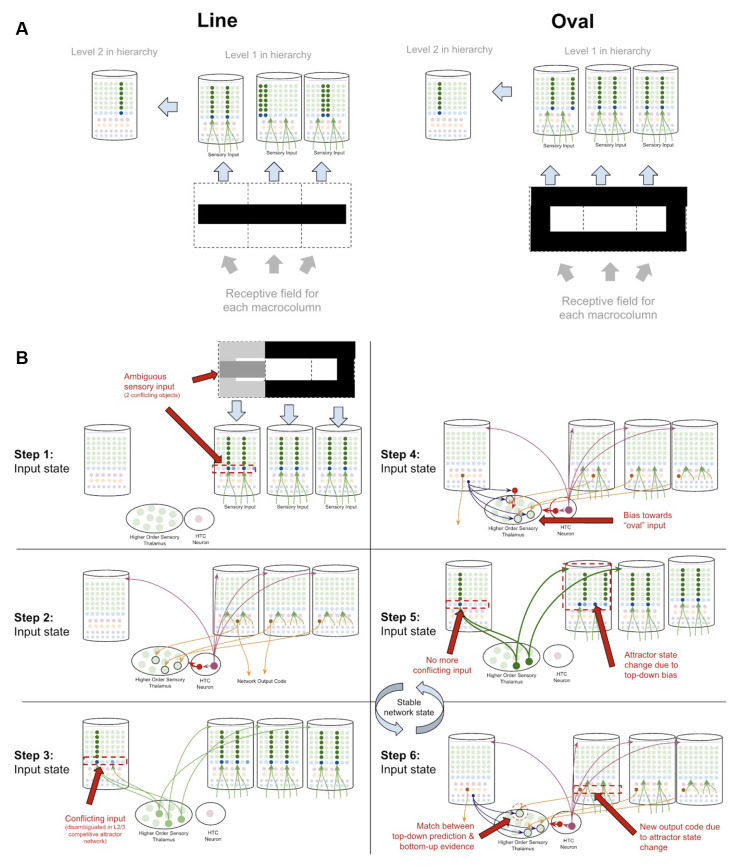
How networks of macrocolumns may perform object disambiguation. **(A)** Depiction of how two visual objects (a line and an oval) might be depicted in a 2-level network of four macrocolumns. The receptive field of each level 1 macrocolumn is depicted by the dotted line squares below each macrocolumn. The inputs to each receptive field for the “line” and the “oval” are shown within the dotted line squares. The patterns activated by the sensory input within each receptive field are shown in the level 1 macrocolumns for line and oval. The macrocolumn in level 2 receives input from all three level 1 macrocolumns through the thalamus (not depicted) and represents the whole object by responding to coincident patterns across the three level 1 macrocolumns. This is consistent with known neuroanatomy - the visual cortex is organized retinotopically. **(B)** Using the model of the two objects (“line” and “oval”) in **(A)**, this figure shows the step by step process by which the network of macrocolumns can disambiguate between the two objects when receiving conflicting sensory input within one receptive field. All macrocolumns in this network are oscillating between “input states” and “output states” at the same time—each step depicted represents an oscillatory phase of the network. See text for details.

Object disambiguation occurs very similarly to sequence disambiguation, except instead of disambiguating between sequence codes, networks disambiguate between conflicting inputs. Suppose a two-level network is learning the difference between an oval and a line, and that each level 1 macrocolumn receives input from only a specific location in a visual field. The learned pattern representations can be seen in Figure 11A, where each level 1 macrocolumn only learns to recognize the shapes seen in its receptive field, while the level 2 macrocolumn can recognize the entire shape by integrating sequence outputs across the level 1 macrocolumns.

What happens then if one of the three level 1 macrocolumns is receiving *conflicting* input? The same dynamics in sequence disambiguation will play out where the competitive network in level 2 will lead to top-down bias in the macrocolumn with conflicting input, eventually leading the entire network to actually see the full oval, even though not all input is consistent with the oval just most of the input (see Figure 11B). This mechanism works mathematically the same way as described in George and Hawkins (2009).

### Multi-areal Matrix Neurons in Thalamus Signal Failed Predictions

A key piece missing from the above proposal is understanding how thalamocortical networks deal with failed vs. successful predictions. There are three reasons why explaining this is essential. First, if a learned sequence fails to predict subsequent input, then a macrocolumn must somehow subsequently “forget” the prior sequence so it can try to look for a new sequence to match with the input it is receiving. In other words, if a macrocolumn knows the sequence “ABC” and “XYZ” if it hears “ABCX” it needs to shift from the “ABC” sequence to the “XYZ” sequence after the surprising “X.” Second, the brain needs to solve the “stability-plasticity dilemma” (Grossberg, 1980)—it is essential that the brain primarily only learns when there is something new to learn, otherwise the brain risks catastrophic forgetting by an overzealous generalization of already learned associations. This requires that the brain has a signal for novelty to modulate the rate of learning. Third, it has been clearly shown through behavioral experimentation that a “surprise” signal is available in the brain. It has been shown that surprise is arousing, that it draws attention, that it dilates pupils, and much more (Itti and Baldi, 2009; Preuschoff et al., 2011). This means that if macrocolumns are in the business of predicting sensory input, then somehow other brain systems become aware of when these predictions are *wrong*, hence there must be some source of failed prediction signal.

I postulate that the answers to all three of these can be found in theoretical work by Stephen Grossberg over 30 years ago in his ART (Grossberg, 1980; Grossberg and Versace, 2008). He proposed that the thalamic core and matrix neurons respond differently when there is a “match” between layer 6a-CT predictions and bottom-up input vs. when there is a “mismatch.” A “match” means that the pattern of core neurons subthreshold activated by L6a-CT are exactly, or close to, the same as the neurons that get activated by the subsequent bottom-up input. He suggested that this double input to core neurons leads them to fire rapidly and oscillate in the gamma frequency. Consistent with this, it has also been shown that if thalamic relay neurons are held at elevated subthreshold resting potentials, they burst fire in response to stimulation, otherwise, they tonically fire in response to stimulation (Jahnsen and Llinás, 1984; Hughes et al., 1999; Sherman, 2001; Guillery and Sherman, 2002). This rapid firing in core relay neurons would then lead to lateral inhibition of matrix neurons through the TRN, hence *reducing* the activity of matrix neurons. In contrast, if there is a mismatch, then core neurons do not fire rapidly when they receive their driving input, hence disinhibiting matrix neurons and *increasing* their activity. Put simply, his matrix neurons signal failed predictions. Consistent with this proposal, it has been found that areas of thalamus rich in matrix neurons, such as the central medial nucleus, respond selectively to unexpected sensory stimuli (Matsumoto et al., 2001; Minamimoto and Kimura, 2002).

**Figure 12:**
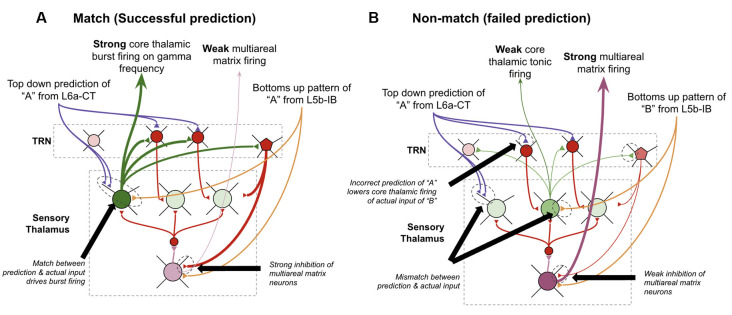
How thalamus may signal failed predictions. **(A)** How thalamic relay neurons respond when top-down predictions from L6a-CT neurons in higher-order macrocolumns successfully match the bottom-up input from L5b-IB neurons in lower-order macrocolumns. Core relay neurons burst fire, and multiareal matrix neurons weakly fire. **(B)** How thalamic relay neurons respond when top-down predictions from L6a-CT neurons in higher-order macrocolumns fail to match the bottom-up input from L5b-IB neurons in lower-order macrocolumns. Core relay neurons weakly tonically fire, which disinhibits multiareal matrix neurons, hence signaling a “failed prediction.” See Figure 2 for neuron types depicted. See text for further details.

I propose that the matrix neurons that fulfill this role are specifically the multiareal matrix neurons recently described in Clascá et al. (2012). Furthermore, I propose that the mechanism for this lateral inhibition of core neurons occurs through the recently elucidated different connectivity of the PV and SOM interneurons within TRN. It was identified that L6a-CT neurons project only back onto PV neurons which seem to subsequently inhibit only core relay neurons. In contrast, SOM interneurons seem to only inhibit matrix neurons and only receive thalamic input *via* lateral inhibition from other relay neurons but not from L6a-CT neurons (Clemente-Perez et al., 2017). Note that this study elucidated PV/SOM interneuron connectivity with specific vs. nonspecific thalamic nuclei as opposed to specifically connectivity with core vs. matrix neurons within specific sensory thalamic nuclei, as I propose in the above model. Hence these studies are only suggestive of selective PV inhibition of core thalamic neurons vs. SOM inhibition of matrix thalamic neurons. However, I believe this is a reasonable extrapolation since the primary distinction between specific and non-specific thalamus is its relative quantity of core vs. matrix neurons (Clascá et al., 2012). Further, this study shows *some* connectivity of SOM neurons in specific thalamus, where there are *some* matrix neurons, but no connectivity of PV neurons with non-specific thalamus, where there are no core neurons (Clemente-Perez et al., 2017). Furthermore, if this ends up not being the case, the above model could be modified to have mismatch codes be signaled from nonspecific thalamic nuclei directly, instead of from multiareal matrix neurons within specific thalamic nuclei (as modeled in Grossberg and Versace, 2008). This observed circuitry provides new experimental support for the above mismatch computation because it implies that the level of inhibition that multi-areal matrix neurons receive depends primarily on the firing rates of core relay neurons. See Figure 12 for details on the proposed circuitry of mismatch signaling.

I propose this mismatch code signaled by multiareal matrix neurons serves three key computational purposes within macrocolumns. First, it resets sequences within L2/3-PY neurons by synapsing directly on inhibitory interneurons in L2/3. Multiareal matrix neurons are known to project to L2/3 of the higher-order cortex, although it is speculative that they synapse on inhibitory interneurons instead of pyramidal cells. By rapidly inhibiting neurons in L2/3, matrix firing makes it such that any L5a-RS sequence biasing within L2/3 is lost, hence restarting any sequences. Second, this mismatch signal generates widespread arousal capable of drawing attention from the frontal cortex. There are several mechanisms through which such arousal could be generated, the simplest being possible direct projections from multiareal matrix neurons to neuromodulatory arousal areas that release acetylcholine or norepinephrine, as is observed from areas of thalamus rich in matrix neurons (Van del Werf et al., 2002). Third, the rapid gamma oscillations in core thalamic neurons generated during “match” episodes generate gamma oscillations within L4-ST and L2/3-PY neurons. These gamma oscillations generate rapid short-term STDP, enabling a rapid acceleration in the rate of learning under conditions of successful predictions. This provides a potential solution to the “stability-plasticity dilemma.”

In Grossberg’s theory, he proposes that the brain can modulate its “sensitivity” to mismatch, a parameter he called “vigilance.” In other words, the brain can decide “how big a mismatch can I tolerate before triggering matrix firing?” Such a mechanism is consistent with the model proposed here, although I do not propose an exact mechanism by which this happens.

## How Networks of Macrocolumns Learn New Objects and Sequences

Now we have all the computational building blocks to answer a key question we set out to answer: how does a macrocolumn learn sequences over realistic timescales, and then output sequence *predictions* to other regions?

To explain this, let us consider a simple procedure—let us see how our model macrocolumn can learn the sequence “ABC” over realistic timescales and once learned, how it can send an output prediction of this sequence “ABC” to other regions after only receiving the sensory input “A.” In our model macrocolumn, let us represent these different elements (“A,” “B,” “C”) by the activation of different sets of two minicolumns (see Figure 3A).

Computationally five specific states will occur during the example procedure of learning this sequence:

(1) Receiving the input of “A” for 1 s:
(2) Pause (no input) for 5 s:
(3) Receiving the input of “B” for 1 s:
(4) Pause (no input) for 5 s:
(5) Receiving the input of “C” for 1 s.

In order to accomplish this task, the brain must store each of these elements in working memory. As such, I propose that the relevant networks lock into an “attentive processing” mode during this procedure. They do this to enable coordinated processing with the frontal cortex and hippocampus, hence oscillating at the theta rhythm. In Supplementary Figure S2 you can see a visualization of this realistic learning procedure, with embedded theta oscillations and the corresponding macrocolumn states.

### Step #1: Receiving Input of “A” For 1 s

**Figure 13:**
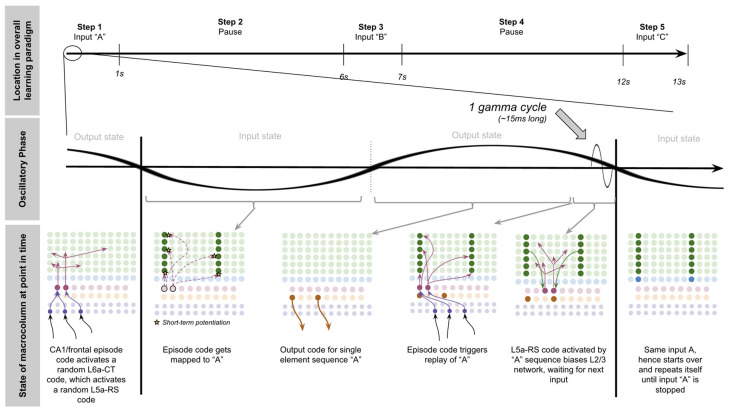
Step 1 of learning paradigm whereby a macrocolumn learns a sequence of elements separated by realistic delays. Top row depicts location in the overall learning paradigm described in text and in Supplementary Figure S2. The middle row depicts the theta phase and gamma phase of the macrocolumn. The bottom row depicts each state of the macrocolumn throughout one full theta cycle within step 1 of the learning procedure. See text for details.

Figure 13 provides a zoom in on the exact representation and processing of the neural circuits in a given macrocolumn during step #1 of this learning procedure. When “A” is input into macrocolumn, the L2/3 pattern for “A” gets activated during the “input state.” When the first “output state” is triggered, a pattern separated L5b-IB representation of the single element sequence “A” gets output. After being initially triggered from the prior activation of “A” in L2/3, the L5b-IB representation turns off any further L2/3 activation (by activating L6a-CT neurons, which then inhibit L4-ST neurons, as described earlier). However, I propose the L5b-IB representation is capable of maintaining itself independently for the duration of the output state, even when the L2/3 state is turned off Harris and Mrsic-Flogel (2013) shows the self-sustaining activity of L5b-IB neurons.

During the output state, I propose the frontal cortex and/or CA1 activates a unique pattern of L6a-CT neurons, which gets mapped to the network state “A” through STDP. I further propose that CA1 generates unique “episodic memory codes,” consistent with the concept of place cells (O’Keefe, 1979) and that frontal cortex propagates a version of these codes to the sensory cortex. If this is the case, then CA1 and frontal cortex would be able to activate and maintain specific memories across the sensory cortex simply by replaying these episode codes. It is of course also possible, and perhaps more likely, that frontal cortex and CA1 replay multiple place codes within a theta cycle. However, for the simplicity of modeling, I will assume that the frontal cortex continuously replays only one “episode code” every theta cycle. This means that every theta cycle, the same unique L6a-CT pattern will be replayed, and hence “A” will be replayed.

After the first replay of “A,” a new L5a-RS code is activated, sequence biasing L2/3-PY neurons. If the subsequent input in the input state is the same (i.e., still just “A”), then the L5a-RS projection fails to change the L2/3 representation. In contrast, if the subsequent input in the input state is different (i.e., it is “B”), then the combination of L5a-RS sequence biasing and L4-ST input will generate a sparse representation in L2/3 unique for the sequence “A→B.”

### Step #2: Pause For 5 s

When the sensory input of “A” is removed during the pause, as long as the frontal/CA1 episode code continues to replay itself during this delay period, then the L6a-CT episode code will continue to independently replay “A” during each output state. Crucially, this means that at the beginning of each input state. L2/3 is sequence biased from the L5a-RS “A” representation, waiting to be mapped to the next incoming L2/3 representation. I propose that this continuous replay of an episode code is one of the key underlying computational processes performed by the brain during working memory tasks.

### Step #3: Input “B” For 1 s

**Figure 14:**
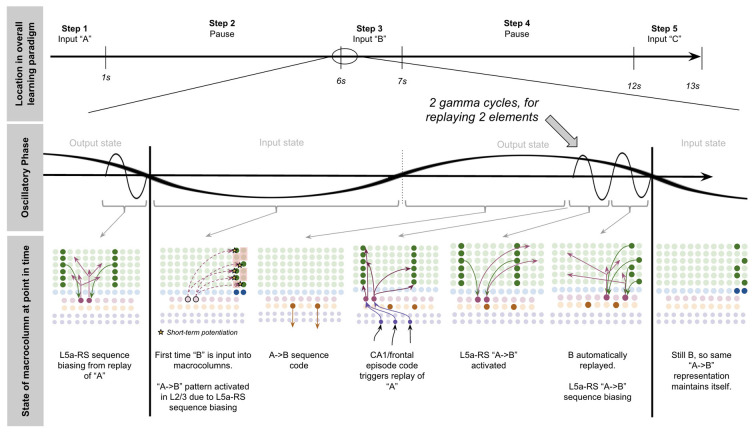
Step 3 of learning paradigm whereby a macrocolumn learns a sequence of elements separated by realistic delays. Top row depicts location in the overall learning paradigm described in text and in Supplementary Figure S2. The middle row depicts the theta phase and gamma phase of the macrocolumn. The bottom row depicts each state of the macrocolumn throughout one full theta cycle within step 3 of the learning procedure. See text for details.

After 5 s of a pause, the sensory input of “B” is provided to the macrocolumn. Due to the sequence biasing from L5a-RS neurons, a sparse representation of “B” is activated that is unique to “A→B,” and the L5a-RS code is mapped to this sparse representation of B using STDP. Due to this unique representation of “B,” now L5b-IB neurons output an “A→B” sequence code instead of just the “A” sequence code (see Figure 14).

Taken together, this means that although “A” and “B” were separated by 5 s, in the macrocolumn they were only separated by ~10 ms due to the repeated working memory replay of “A.” This enables rapid STDP plasticity between the L5a-RS neurons activated by “A” and the L2/3 representation of “B,” despite a 5-s separation between the actual sensory stimuli.

When the repeating frontal/CA1 episode code comes around and reactivates “A” during the output state, the entire sequence “A→B” will be replayed automatically, instead of just “A.”

### Step #4: Pause For 5 s

Due to the same dynamics described in step #2, as long as frontal cortex/CA1 continues to replay the same episode code, our model macrocolumn will continue to replay the sequence “A→B” on each output state even when stimuli “B” is removed.

The key difference between step #4 and step #2 is that now: (a) there are two elements replayed and hence two gamma cycles (A and then B); and (b) the output state now ends with a sequence bias from the L5a-RS code for “A→B,” instead of the L5a-RS code for just “A.”

### Step #5: Input “C” For 1 s

**Figure 15:**
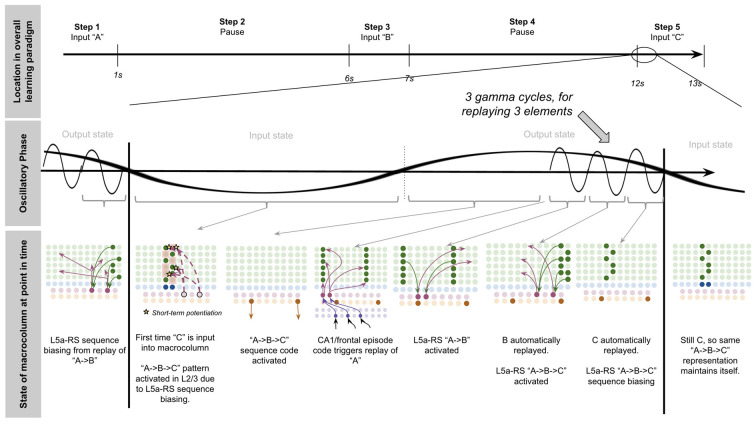
Step 5 of learning paradigm whereby a macrocolumn learns a Sequence of elements separated by realistic delays. Top row depicts location in the overall learning paradigm described in text and in Supplementary Figure S2. The middle row depicts the theta phase and gamma phase of the macrocolumn. The bottom row depicts each state of the macrocolumn throughout one full theta cycle within step 5 of the learning procedure. See text for details.

When “C” is finally inputted into the macro column after the final 5-s interval, as in step #3, the sequence bias from L5a-RS code for “A→B” leads to a sparse representation of “C” that corresponds to the sequence “A→*B*→C” (see Figure 15). This builds plasticity between the L5a-RS code for “A→B” and this sparse representation of “C.” Hence now when “A” is replayed during the output state, there will be 3 elements replayed (hence 3 gamma cycles): “A” then “B” then “C.”

During the output state, due to the L2/3 representation of “C” that is unique to “A→*B*→C,” the L5b-IB output code will now be a unique code that represents exactly the sequence “A→*B*→C”

This macrocolumn has accomplished something amazing—it is now outputting a unique sequence code for the sequence “A→*B*→C” even though the input elements were separated by long time intervals. And the only external computation required was a constant episode code from the frontal cortex and/or hippocampus to enable consistent replay of only the* first* element “A.”

### Remembering the Sequence “ABC” After Just Saying “A”

Each time the sequence “A” then “B” then “C” replays during an output state while L5b-IB neurons are firing the “A→*B*→C” output code, each representation of “A” then “A→B” and then “A→*B*→C” builds plasticity with L5b-IB representation of “A→*B*→C” (since they coactivate with each other). If this replay occurs a sufficient quantity of times, these synaptic connections will go through long-term potentiation (LTP). This LTP then makes it such that when this macrocolumn receives the input “A,” during the output state it will output the code “A→*B*→C” *automatically* instead of just the output for sequence “A.” Note that multiple L5b-IB representations can be active simultaneously, meaning that if “A” leads to multiple different sequences, multiple ambiguous sequence codes can be output for higher cortical areas to disambiguate.

## Discussion

This aricle provides a novel theory for how the neocortex learns to recognize complex multi-sensory objects and sequences across realistic timescales, and in doing so provides a model for how working memory may function. It extends prior HTM models in three ways: (1) it shows how macrocolumns perform working memory and connect sequences separated by long time intervals; (2) it shows how networks of macrocolumns can coordinate processing with other macrocolumns on precise time intervals; and (3) it explicitly incorporates top-down attention.

Taken together, this sheds light on the overall evolutionary purpose of the mammalian neocortex. As theorized by Hawkins’ HTM, I propose the neocortex is in the business of generating unique sequence and object codes. This neocortical microcircuit engages in “unsupervised learning.” There is no labeling. The only “supervisor” is *time* and *attention*. Inputs that tend to co-occur, or to quickly follow each other in sequence, will over time get unique output codes from a network of macrocolumns. These unique sequence codes can then be used by other structures, such as the striatum, amygdala, or other cortical areas to respond to recognized objects or sequences.

In addition to explaining *how* working memory, sequence prediction, and object recognition might work in the neocortex, this theory also explains *why* certain neuroscientific findings are observed. I will review a selection of these below.

## An Explanation for Why We Observe Network Oscillations Within the Neocortex and Thalamus

It has long been observed that there are distinct electrophysiological oscillations within the brain. However, the computational purpose has been unclear. Many computational models have attempted to explain these oscillations as emergent dynamics of feedback inhibition and attractor states (Lundqvist et al., 2011; Mejias et al., 2016). Other theories have suggested that oscillations serve the specific purpose of entraining distributed networks to process together (Lisman and Jensen, 2013; Doesburg et al., 2015; Ribary et al., 2019). Consistent with the latter proposal, the theory presented here provides a circuit level model for how oscillations arise and why they are essential for distributed processing within the neocortex. Specifically, this theory suggests that the purpose of these oscillations is to coordinate input and output states across networks of macrocolumns to enable integrated processing on precise time scales.

## An Explanation for Why We Observe Working Memory to Cap Out at ~7 Items

sychologist George Miller showed that the average human can only hold around seven items in short-term working memory at a given point in time (Miller, 1956). However, a neural circuit explanation for *why* we have this working memory limitation has been elusive. Lisman and Idiart (1995) made the novel observation that the two frequencies observed in EEGs during working memory tasks, theta and gamma oscillations, have a clear relationship with the “magic number 7”: there are ~7 gamma oscillations within one half of a theta wave (~100 ms). They went on to propose that elements in working memory are replayed at the gamma frequency every theta cycle (Lisman and Idiart, 1995; Jensen and Lisman, 2005).

Consistent with their idea, I propose that the reason we have this limitation is that the thalamocortical networks provide a maximum of ~100 ms within an output state for elements to be replayed on the gamma frequency. Hence macrocolumns only have the time to replay ~7 elements; anything more simply will get truncated when the next input state comes around. A reasonable follow-up question would then be “why can not the output state simply be made *longer* to support more than 7 elements?” The answer may lie in the fact that extending the length of output states is not free. If working memory operates works as proposed in this theory, then there is a costly tradeoff between working memory capacity and the speed of processing—the longer the output state, the more items that can be held in working memory, but the *slower* the processing of incoming sensory data will be passed up and down macrocolumn hierarchies. I hypothesize that there is an evolutionary reason why the human brain has settled around ~7 being the point at which the additional slowness in processing is no longer worth the benefit to increased working memory capacity.

## An Explanation for Why We Observe Mammals Without a Hippocampus to be Impaired at Sequence Memory and Unable to Create New Memories

Patients with bilateral damage to their hippocampus lose the ability to produce new episodic memories, but can still remember old memories (Parkin, 1996; Corkin, 2002), but it has not been clear exactly why this is the case. This model explains this observation: the hippocampus is a key source (likely disynaptically through frontal cortex) of memory reactivations within the neocortex. I hypothesize that this replay is required for new episodic memories to be learned. Without this frontal/CA1 episode code, a replay will not occur reliably in the neocortex. Without these repeated replay events, memories can never transition from short-term potentiation to LTP. In contrast, memories that are already present in LTP of synapses can still be recalled with the right sensory input.

Furthermore, patients with hippocampal damage also have profound deficits in specifically sequence memory (Agster et al., 2002), while other forms of intelligence and object recognition remain functional (Honey et al., 1998; Jensen and Lisman, 2005). The sequence memory deficit occurs most notably when there are *time delays* incorporated into the learning paradigms (Agster et al., 2002). These results can be explained with the model presented here: without a consistent frontal/CA1 episode code, sequence elements can not be maintained in working memory, hence a sequence provided over realistic timescales will never be learned. On the other hand, hierarchies of macrocolumns will still be able to engage in object recognition of known objects, since there is no requirement for replay events.

## An Explanation for How the Brain May Generalize Object Recognition to Changes in Orientation, Translation, and Scale

The proposed circuitry potentially provides a clue as to how to solve a vexing problem in machine vision research—learning to generalize recognition well to changes in orientation, translation, and scale (Hassabis et al., 2017). You can show a human a shape at one level of scale, and in one shot, a human will recognize that same object when perturbed, shrunk, or shifted to another field of view (Carey and Barlett, 1978). However, in machine vision, this level of generalization has not been achieved. The state-of-the-art way to solve this problem is to artificially edit the training data to include changes in orientation, translation, and scale for a given object. Obviously, the brain does not require this. The proposed L6-CC to L6a-CT circuitry provides a clue as to how the brain might solve this problem. The brain may do more than simply learn to associate a set of inputs with a specific label (say a “cat”). The sensory cortex may first learn that a 45-degree bar over here is the same thing as a 45-degree bar over there. In other words, the brain learns the relationship of low-level features to movement. This may be how the brain in one shot can recognize that an object at one level of scale is the same object at another level of scale, translation, or orientation. There have been notable mathematical attempts to resolve this type of one-shot learning (Ranzato et al., 2007; Lake et al., 2011; Doumas et al., 2018), some of which implement a type of low level “feature invariance” similar in concept to the L6-CC to L6a-CT transfer network proposed here.

## Is Neocortex Actually Uniform?

There are legitimate challenges to the hypothesis that the neocortex implements a repeated canonical microcircuit. The theory here focused solely on modeling the canonical microcircuit within the sensory cortex since there is meaningful evidence that the frontal cortex performs different computations than the sensory cortex. However, even in sensory cortex, there are meaningful interregional differences in laminar widths (Fukutomi et al., 2018), and evidence that layers exhibit different correlated variability profiles across different regions, implying differences in canonical circuits (Hansen et al., 2012; Nandy et al., 2017). I propose that differences in width can be explained simply by variances in computational load within the same cortical microcircuit. For example, if in certain domains there is lots of motor input to be integrated, you may expect a uniquely thick L6, such as what you observe in the parietal cortex (Fukutomi et al., 2018). However, differences in correlated variability are harder to explain in the context of this theory. Further work will have to be done to reconcile this observation with the theory presented here.

## The Mystery of Bottom-Up Thalamic Input to Layer 5b Intrinsically Bursting Neurons and Potential Modifications of This Theory

Although this model explains a broad set of experimental findings and assigns a function to a broad set of the observed connectivity within a macrocolumn, a key experimental finding that is directly inconsistent with the model presented here is the observed direct bottom-up thalamic input to L5b-IB neurons (Constantinople and Bruno, 2013). First, it has been shown that L5b-IB neurons fire *before* L4-ST neurons when receiving sensory stimuli (Constantinople and Bruno, 2013; Sun et al., 2013), whereas this model would predict it should fire last. Second, it has been found that L5b-IB neurons are insensitive to L4-ST inactivation (Constantinople and Bruno, 2013).

I hypothesize two solutions to this. One solution could be that after sufficient training, L5b-IB neurons learn to respond directly to thalamic input, learning to predict the current sequence-based solely on the inputs alone. The benefit of this would be faster processing. Another solution could be that both L4-ST and L5b-IB are parallel coincident detectors of bottom-up input, and sequence representations in L5b-IB emerge only through learned L2/3-PY modulation of L5b-IB representations. If the latter ends up being true, then this model will have to be modified to provide a different mechanism for how sequences get learned in L5b-IB neurons.

## Testable Predictions

Many of the predictions of this theory are consistent with the general predictions of HTM, which can be seen in Hawkins and Ahmad (2016). However, in addition to these, several predictions are specific to the theory presented here:

1. This theory suggests that multiareal matrix neurons signal “mismatches.” A way to test this would be to record individual multiareal matrix neurons during the presentation of both surprising and well-learned stimuli. This theory predicts that multiareal matrix neurons will selectively burst in response to surprising stimuli (i.e., “mismatch” responses).
2. This theory predicts that “multiareal matrix neurons” seen in Clascá et al. (2012) are the same as the “high threshold bursting” thalamocortical relay neurons seen in Lörincz et al. (2009) and Hughes et al. (2011). Tracing studies on HTC neurons will elucidate whether or not this prediction is correct.
3. This theory suggests that when multi-areal matrix neurons burst fire, they “restart” sequence representations stored within macrocolumns. A way to test this would be to selectively inhibit multi-areal matrix neurons during tasks where subjects are observing well-learned sequences (moving objects, melodies, etc.). This model predicts that in such a condition if surprising stimuli are inserted into these sequences, it will take subjects longer to identify these new objects. This model further predicts that subjects will struggle to identify which stimuli are in fact “surprising” at all.
4. This theory suggests that attention facilitates learning of sequences by replaying previously received elements through the activation of L6a-CT neurons. A way to test this would be to selectively inhibit frontal and hippocampal input to L6a-CT neurons during learning tasks. This model predicts that subjects should be severely impaired in their ability to learn sequences of stimuli if the presentation of these stimuli is separated by a delay.
5. This theory suggests that intended motor movements arise through frontal projections to L6a-CT neurons. A way to test this would be to selectively inhibit frontal input to L6a-CT neurons within the visual cortex. This model predicts that this will be very disorienting for subjects as they will be unable to use intended volitional movements to predict changes in visual stimuli. This difference could be tested using measures of arousal and surprise.
6. This theory suggests that the origin of differences in observed responses within L2/3 from predicted vs. surprising stimuli (Jordan and Keller, 2020) arises from multiareal matrix neurons inhibiting L2/3-PY neurons in response to surprise. A way to test this would be to selectively inhibit matrix neurons within sensory thalamus. This theory predicts that in such a condition, prediction error responses within L2/3 of the corresponding area of neocortex would be eliminated.
7. This theory suggests that L6-CC neurons pass lateral predictions of upcoming sensory stimuli in response to volitional movement. A way to test this would be to selectively inhibit the L6-CC neurons within the receptive field of visual stimuli a subject is directed to saccade towards. This theory predicts that subjects will be selectively unable to predict input from the inhibited visual field while being able to predict input from other visual fields normally.

## Relationship to Previous Models

### Relationship to Previous Models of Hierarchical Temporal Memory

The model presented here is highly inspired by the HTM model presented by George and Hawkins (2009). I maintain the key elements of their model while extending it to support learning over realistic time scales, coordination between macrocolumns, working memory, attention, and motor predictions. To accomplish this, there are some key differences in terms of the actual microcircuits presented. Previous models of the canonical microcircuit typically involve a linear computation from L4 as the input layer, L2/3 as the processing layer, and L5/6 as the output layer. However the observed connectivity between layers is far more complex: the layer 5 “output layer” sends massive projections back to L2/3 (Dantzker and Callaway, 2000; Adesnik and Naka, 2018), L5 is massively horizontally reciprocally connected (Naka and Adesnik, 2016), L6a-CT cells send a strong projection to L4 inhibitory GABA interneurons and L5a-RS neurons (Thomson, 2010; Kim et al., 2014). The theory presented here explains the computational function such circuitry may provide.

The model presented here is also highly inspired by the Hawkins and Ahmad’s (2016) model of sequence memory. Hawkins and Ahmad (2016) proposed a novel way in which sequence codes could be remembered through sparseness in minicolumns. Four open questions (my assessment) from their article were: (1) how “timing” of processing is coordinated with other macrocolumns; (2) how these “sequence predictions” get communicated *outside* of a macrocolumn; (3) how sequences get learned when there is a realistic time delay between inputs (seconds to minutes); and (4) how sequences get “restart.” I extend Hawkins’ article by proposing an explanation for all four. A notable difference in the circuit model presented here vs. Hawkins and Ahmad (2016) is that in this model, sparseness in minicolumns is achieved *via* “sequence bias” from L5a RS neurons projecting to L2/3, whereas in their model it occurs *via* lateral connections of L2/3 pyramidal cells. The model presented here proposes that the lateral connectivity of L2/3 pyramidal cells instead plays the role of implementing a competitive network, instead of making sequence predictions. Another difference is that their model proposes “restarting” occurs through L4 stellate cells “overriding” sparse representations when incoming input is “unpredicted,” although they acknowledge a lack of a circuit explanation for how this would occur. This model proposes sequence restarting occurs through the toggling of input states to output states.

Hawkins et al. (2019) and Lewis et al. (2019) provide a compelling theory that L6 contains “grid-like” neurons that can perform transformations on incoming sensory input to L4 using movement signals, enabling stable L2/3 representations despite changes in sensory input (i.e., generating allocentric representations of objects). The model presented here is broadly consistent with their proposal, albeit with several extensions. First, this theory presents an explicit neural circuit model for how the neocortex performs these functions, assigning computational roles to known categories of neurons. Second, while their model shows how a single macrocolumn can learn to recognize an object, the theory presented here incorporates long-range macrocolumn-to-macrocolumn connectivity, enabling networks of macrocolumns to learn objects hierarchically and transfer representations laterally.

Last, prior HTM models do not explicitly model surprise, and hence fail to explain behaviorally observed responses to surprise, as well as do not explicitly model modulations of learning rates during successful vs. failed predictions. This presents a problem in the context of the stability-plasticity dilemma—supervised systems will risk learning “too much” or over-generalizing. Mathematically, HTM models resolve this by assuming that inactive neurons get “boosted” whereas highly active ones get suppressed (Hawkins et al., 2010)—I am unaware of experimental support for such a mechanism. In contrast, this theory suggests that thalamic oscillations and attention selectively gates learning.

### Relationship to Previous Models of Working Memory and Delay Activity

There are three broad classes of computational models for working memory and delay activity: (1) attractor network models; (2) bistability models; and (3) synaptic weight models (Sreenivasan and D’Esposito, 2019). Stable attractor network models and bistability models both rely on persistent representations maintaining themselves during working memory delay periods. Because of this, both of these models struggle to explain how: (a) multiple items can be stored in working memory simultaneously; and (b) how working memory can be maintained without disrupting ongoing sensory processing.

On the other hand, synaptic weight models propose that working memory is stored in short-term synaptic potentiation after stimuli are received, instead of within an actual persistent representation. These models are attractive because they do not disrupt incoming sensory information and theoretically could support multiple items being stored simultaneously. However, synaptic weight models have struggled to: (a) explain the observed delay activity in the brain during working memory tasks; and (b) describe a circuit mechanism for multi-item storage.

This model proposes a “synaptic weight model” that solves both of these challenges. This model explains the existence of delay activity as well as how multiple items can be stored simultaneously (through “replays”), all without the requirement for a stable attractor state.

### Relationship to Previous Models of Predictive Coding and Active Inference

Predictive coding is undeniably the most broadly accepted computational model of the sensory cortex. The canonical model of predictive coding assumes that hierarchical layers sensory neocortex pass predictions to lower level neocortex, and pass prediction errors up to higher levels (Rao and Ballard, 1999; Bastos et al., 2012; Spratling, 2017; Keller and Mrsic-Flogel, 2018). There are notable similarities between the model presented here and predictive coding. Top-down modulatory input in this model and other HTM models is functionally similar to the top-down control of Kalman gain or precision in predictive coding. Attempts to extend predictive coding to incorporate temporal dynamics, such as those that simulate birdsong (Kiebel et al., 2009a,b; Isomura et al., 2019), also share several features with HTM models. First, such models propose that recognition occurs through multiple internal generative models each attempting to predict its sensory input, leading the network to eventually select the model that provides the most plausible explanation for its input. This is conceptually very similar to the model here whereby winner-take-all dynamics amongst L2/3-PY neurons force only the representation with the most bottom-up evidence to become activated. Second, they model the ability for an agent to switch between different generative models to perform “hypothesis testing,” a dynamic conceptually similar to this model’s top-down attention, whereby ambiguous input can be disambiguated through top-down bias towards specific representations.

Continuous state-space models of attention and working memory (Parr and Friston, 2017) also share similarities to the model presented here. In those models, a key function of working memory is to enable a serial sequence of evidence to accumulate over time to select the internal generative model with the most evidence. This is conceptually similar to the process here whereby the context of prior stimuli is maintained within macrocolumns, allowing subsequent sensory input (new “evidence”) to further disambiguate the sequence representation. Interestingly, their model used a similar theta cycle of evaluation and broadcasting proposed in this article. In their setting, Bayesian inference proceeds by the minimization of variational free energy (i.e., maximization of marginal likelihood) through attractor dynamics to a free energy minimum, every theta cycle. The output is then broadcast to other levels of deep generative models.

The model presented here is also consistent with Friston’s seminal Free-Energy Principle, a foundational principle guiding most predictive coding models (Friston, 2010). Put simply, Friston’s free-energy principle proposes that the brain seeks to minimize “surprise.” This general principle is consistent with the theory presented here—networks will seek to minimize surprise by resetting representations when they fail to predict subsequent input, and selectively learning new representations only during: (a) successful matching; or (b) attentive processing. It is relevant to note that this synergy between active inference and this model does not extend cleanly to other HTM models—other HTM models do not explicitly model the surprise/non-matching dynamics proposed here. As such, the theory here presents a potential unifying bridge between predictive coding and HTM.

However, despite these many similarities, there are also important differences between the theory presented here and predictive coding. Most directly, predictive coding typically theorizes that L2/3-PY neurons compute prediction errors, and L5/6 neurons compute predictions (Bastos et al., 2012), whereas this model proposes no explicit prediction error computation, but rather a non-negative “mismatch” code signaled by multiareal matrix neurons. The benefit of this mismatch code is that unlike prediction error computations, it does not require signaling negative values, and can enable learning without biologically implausible “weight copying” (for a review of these limitations within predictive coding models see Spratling, 2017).

### Relationship to Previous Models of Adaptive Resonance Theory

ART is built on the idea that the neocortex tries to assign incoming sensory input to a known classification, and if the mismatch between incoming input and all known classifications surpasses a threshold, then a new classification is generated for that input (Grossberg and Versace, 2008). ART shares several features with the theory presented here—first, both propose the neocortex learns in an unsupervised manner, second, both propose that matrix neurons signal mismatch, third, both proposes that L6a-CT neurons signal the top-down predictions from which this mismatch is computed, and fourth both propose that attentional effects are at least partially mediated through L6a-CT neurons. However, this model extends ART in several meaningful ways. Most notably, the ART model does not explicitly model working memory or sequence learning. Attempts to extend ART into these domains have relied on working memory models of attractor networks with order maintained with relative levels of excitation between each element (Grossberg, 1982, 2007). Such a model relies on hard to imagine systems that maintain precise levels of firing rates amongst a distributed network of representations. Furthermore, even these extensions of ART do not explain how a sequence can be stored in long term memory, how overlapping sequences can be disambiguated, nor how learned sequences can be used to predict upcoming input. The model presented here proposes an answer to all of these. Last, ART relies on a relatively implausible mechanism of rapid hypothesis testing that loops through different possibilities before choosing to generate a new cluster. In contrast, the theory presented here suggests that top-down attention and replay mediates this learning of new representations directly.

## Data Availability Statement

The original contributions presented in the study are included in the article/Supplementary Material, further inquiries can be directed to the corresponding author.

## Author Contributions

MB conceived the overall theory and wrote the entire manuscript.

## Conflict of Interest

The author declares that the research was conducted in the absence of any commercial or financial relationships that could be construed as a potential conflict of interest.

## Supplementary Material

The Supplementary Material for this article can be found online at: https://www.frontiersin.org/articles/10.3389/fncir.2020.00040/full#supplementary-material.

FIGURE S1 The duck or rabbit illusion. Example of how top-down bias can change object perception without any changes to bottom-up input. Originally printed in the 1892 issue of *Fliegende Blätter*.

FIGURE S1 Visual depiction of example paradigm for learning a sequence of elements separated by realistic time delays. The top part of the figure shows the timeline of the learning paradigm. The “zoom in” depicts the repeating oscillatory states of macrocolumns during the learning paradigm. See text for details.

TABLE S1 References for connectivity. References for the connectivity modeled and cited in this article.
