# Supplementary figures and images for: An Attempt at a Unified Theory of the Neocortical Microcircuit in Sensory Cortex
Source: Front Neural Circuits. 2020 Jul 28;14:40. doi: 10.3389/fncir.2020.00040 (PMC7416357; doi:10.3389/fncir.2020.00040)

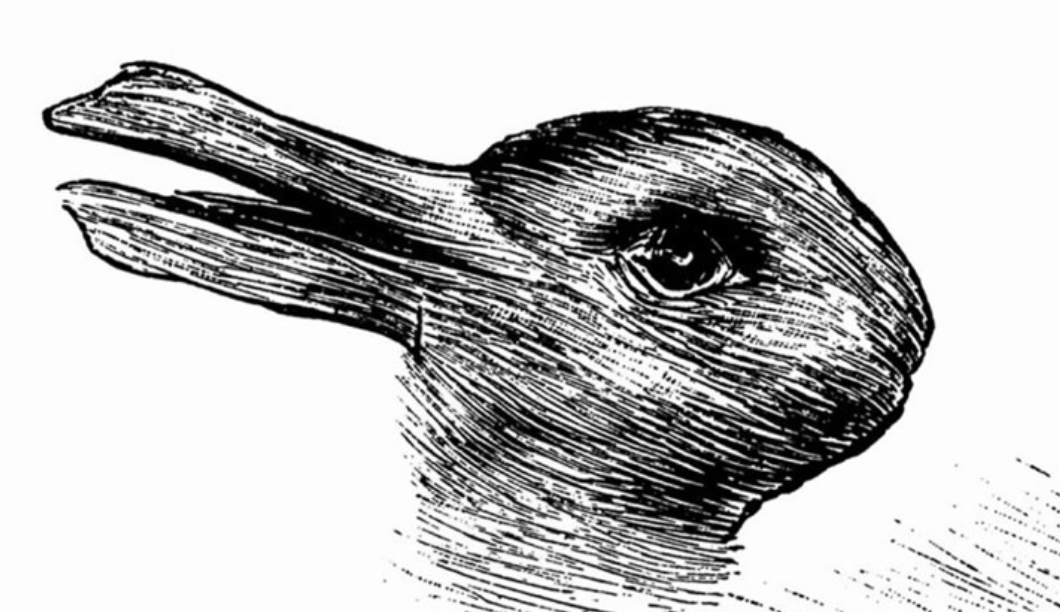

Supplement: FIGURE S1 — The duck or rabbit illusion. Example of how top-down bias can change object perception without any changes to bottom-up input. Originally printed in the 1892 issue of Fliegende Blätter. [file Image_1.jpeg]

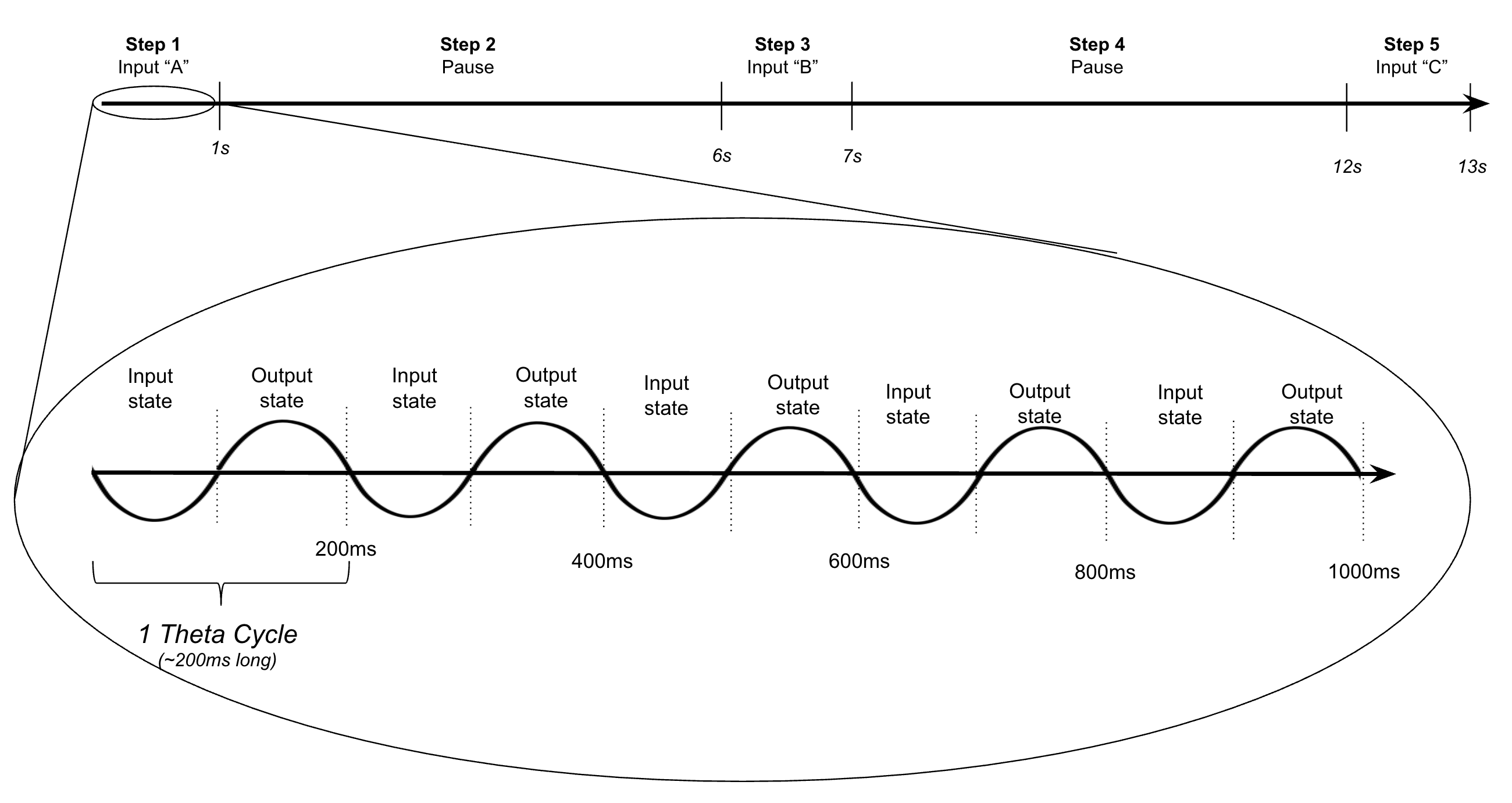

Supplement: FIGURE S1 — Visual depiction of example paradigm for learning a sequence of elements separated by realistic time delays. The top part of the figure shows the timeline of the learning paradigm. The “zoom in” depicts the repeating oscillatory states of macrocolumns during the learning paradigm. See text for details. [file Image_2.jpeg]
