# Supplementary material for: An Attempt at a Unified Theory of the Neocortical Microcircuit in Sensory Cortex
Source: Front Neural Circuits. 2020 Jul 28;14:40. doi: 10.3389/fncir.2020.00040 (PMC7416357; doi:10.3389/fncir.2020.00040)
Supplement: TABLE S1 — References for connectivity. References for the connectivity modeled and cited in this article. [file Table_1.pdf]

Supplementary Information

Table S1: References for Connectivity

Projection	Citations
L2/3-PY -> other L2/3-PY	Bannister, 2005, Markram et al., 2004
L2/3-PY -> interneuron -> other L2/3-PY	Bannister, 2005, Markram et al., 2004
L2/3-PY -> L5a-RS	Larsen & Callaway, 2005, Kawaguchi, 2017, Kampa et al., 2006
L2/3-PY -> L5b-IB	Larsen & Callaway, 2005
L4-ST -> L2/3-PY	Douglas & Martin, 2004
L4-ST -> Interneuron -> L5a-RS and L5b-IB	Pluta et al., 2015, Naka & Adesnik, 2016
L5a-RS -> L2/3-PY	Dantzker & Callaway, 2000; Adesnik & Naka, 2018
L5a-RS -> L2/3 interneurons -> L2/3-PY	Dantzker & Callaway, 2000; Adesnik & Naka, 2018
L5b-IB -> L6a-CT	Zarrinpar & Callaway, 2006
L5b-IB to higher-order relay neurons	Baker et al., 2018, Kim et al., 2015 Deschênes et al. 1994, Rouiller and Welker 2000; Reichova and Sherman 2004; Groh et al. 2008, Theyel et al., 2009, Harris & Mrsic-Flogel, 2013, Sherman, 2017, Llano & Sherman, 2008
L6a-CT -> L6 interneuron -> L2/3-PY	Bortone et al., 2014
L6a-CT -> L6 interneurons -> other L6a-CT	Thompson 2010
L6a-CT -> L4-ST	Thompson 2010, Kim et al., 2014, Ahmed et al., 1994, Binzegger, 2004
L6a-CT -> L4 interneuron -> L4-ST	Thompson 2010, Kim et al., 2014
L6a-CT to lower order thalamic relay neurons	Reichova and Sherman 2004, Thomson, 2010, Sherman, 2017
L6a-CT > TRN neurons	Clemente-Perez et al., 2017
L6-CC -> local L6a-CT and/or L6-CC	Harris & Mrsic-Flogel, 2013
L6-CC -> long range L6a-CT and/or L6-CC	Harris & Mrsic-Flogel, 2013

Core thalamic neurons -> L4-ST	Hegdé & Felleman, 2007
Core thalamic neurons -> TRN -> other thalamic relay neurons	Pinault & Deschênes, 1998
